# CHD7 promotes glioblastoma cell motility and invasiveness through transcriptional modulation of an invasion signature

**DOI:** 10.1038/s41598-019-39564-w

**Published:** 2019-03-08

**Authors:** Raquel A. C. Machado, Hannah Schneider, Carlos DeOcesano-Pereira, Flavio Lichtenstein, Fernando Andrade, André Fujita, Marina Trombetta-Lima, Michael Weller, Christian Bowman-Colin, Mari Cleide Sogayar

**Affiliations:** 1 Department of Biochemistry, Chemistry Institute, University of São Paulo, São Paulo 05508-000 SP, Brazil; 2 Cell and Molecular Therapy Center (NUCEL), Internal Medicine Department, School of Medicine, University of São Paulo, São Paulo 05360-130 SP, Brazil; 3 Laboratory of Molecular Neuro-Oncology, Department of Neurology, University Hospital and University of Zurich, Zurich, Switzerland; 4 Centre of Excellence in New Target Discovery (CENTD), Butantan Institute, São Paulo, SP Brazil; 5 Department of Computer Science, Institute of Mathematics and Statistics, University of São Paulo, São Paulo, Brazil; 6 Dana Farber Cancer Institute, Harvard Medical School, 1 Jimmi Fund Way - SM808, Boston, MA USA

## Abstract

Chromatin remodeler proteins exert an important function in promoting dynamic modifications in the chromatin architecture, performing a central role in regulating gene transcription. Deregulation of these molecular machines may lead to striking perturbations in normal cell function. The *CHD7* gene is a member of the chromodomain helicase DNA-binding family and, when mutated, has been shown to be the cause of the CHARGE syndrome, a severe developmental human disorder. Moreover, *CHD7* has been described to be essential for neural stem cells and it is also highly expressed or mutated in a number of human cancers. However, its potential role in glioblastoma has not yet been tested. Here, we show that CHD7 is up-regulated in human glioma tissues and we demonstrate that *CHD7* knockout (KO) in LN-229 glioblastoma cells suppresses anchorage-independent growth and spheroid invasion *in vitro*. Additionally, *CHD7* KO impairs tumor growth and increases overall survival in an orthotopic mouse xenograft model. Conversely, ectopic overexpression of CHD7 in LN-428 and A172 glioblastoma cell lines increases cell motility and invasiveness *in vitro* and promotes LN-428 tumor growth *in vivo*. Finally, RNA-seq analysis revealed that CHD7 modulates a specific transcriptional signature of invasion-related target genes. Further studies should explore clinical-translational implications for glioblastoma treatment.

## Introduction

Chromatin remodeler proteins comprise a special class of enzymes which modify chromatin structure, thereby playing an essential role in modulating gene expression patterns^1^. The CHD proteins are ATP-dependent chromatin remodelers which utilize the energy from ATP hydrolysis to slide nucleosomes, dissociate core histones, or relocate entire histone octamers^2^. Abnormal activity of these molecular machines may result in a multitude of deregulated cellular programs, affecting cell survival, cell death, or malignant transformation in a cell type-specific fashion^3^.

Over 90% of the patients displaying clinically typical CHARGE syndrome carry mutations in *CHD7*^4^, highlighting the role of this protein in regulating gene expression during tissue development and maintenance. *Chd7*-null mouse embryos lose viability by E11^5^, whereas heterozygozity in mouse, *Xenopus* and zebrafish models recapitulate many of the malformations present in CHARGE patients^6–9^.

Functional studies showed that CHD7 binding sites display features of enhancer elements, predominantly decorated with high levels of mono-methylated histone H3K4 in a cell type- and stage-specific manner^10–12^. In addition, CHD7 cooperates with PBAF (Polybromo-associated BAF) complexes in *Xenopus* neural crest cells to regulate crucial transcription factors, allowing for the acquisition of multipotency and migratory potential^7^. Using both proteomic and genomic approaches, CHD7 was also found to be a transcriptional cofactor of the essential neural stem cell (NSC) regulator, Sox2, suggesting a role for CHD7 in neurogenesis^13^. In this context, CHD7 was shown to be critical for activation of the neural differentiation program of NSC and progenitors in the adult mouse brain^14^ and its inactivation resulted in loss of stem cell quiescence, leading to significant decline in the number of newborn neurons^15^.

Frequent mutations of *CHD7* and/or altered gene expression have been reported in different human cancers^16–19^. CHD7 (formerly known as KIAA1416) has been reported to be up-regulated in colon cancers^20^ and *CHD7* gene rearrangement was suggested to be a driver mutation in small-cell lung cancer^21^. Additionally, low CHD7 expression was associated with improved outcome in patients with pancreatic ductal adenocarcinoma treated with gemcitabine^22^. However, the potential contribution of CHD7 to glioblastoma tumor biology had not yet been tested. Using a candidate gene approach, we set out to investigate a possible role for CHD7 in glioblastoma, given its pivotal role for NSC function and the evidence for CHD7 alterations in other tumor types.

## Results

### CHD7 expression is up-regulated in gliomas

**Figure 1:**
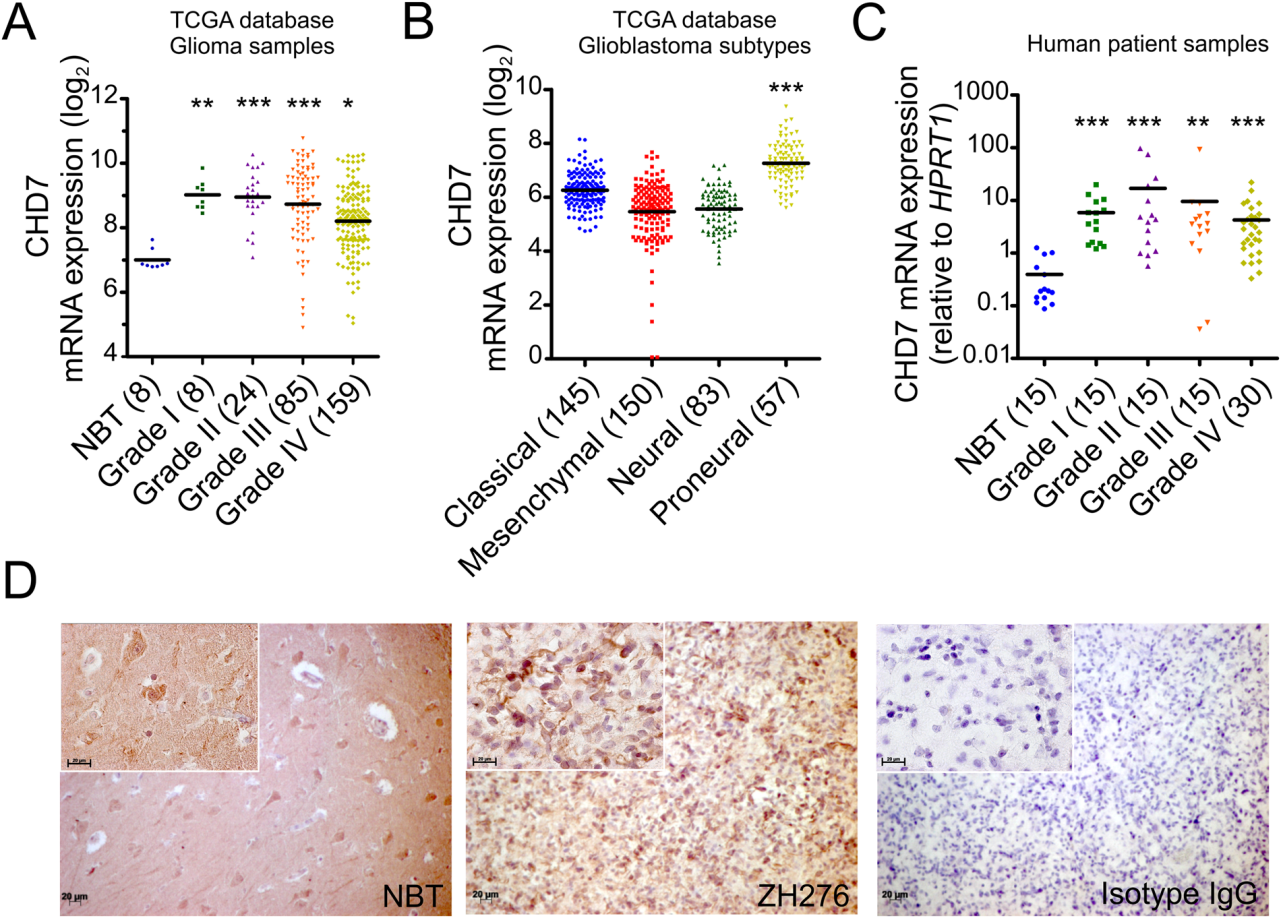
*CHD7* is up-regulated in gliomas. (**A**) CHD7 mRNA levels in 276 human brain tissue samples from the TCGA microarray database. Values are presented as log_2_ transformation gene normalized by median-centered Log_2_ ratios. (**B**) CHD7 mRNA levels in glioblastoma subtypes according to the Verhaak classification. Values are presented as log_2_ transformation gene normalized by median-centered Log_2_ ratios. (**C**) Relative CHD7 mRNA levels of macro-dissected brain tissue samples from normal brain tissue (NBT) and from resected glioma specimens was assessed by qRT-PCR. Values are presented as linear on a logarithmic scale (log10). HPRT1 levels were used as internal control for normalization. Bars represent the mean value. ^*^p < 0.05, ^**^p < 0.01, ^***^p < 0.001; non-parametric analysis of variance (Kruskal-Wallis test) followed by Dunn’s test for post hoc comparison were used for statistical analysis. (**D**) Representative CHD7 immunohistochemistry in NBT and in ZH276 glioblastoma patient sample. Isotype IgG was used as negative control. Scale bar = 20 μm.

To investigate a potential role for CHD7 in human glioblastoma, we first examined CHD7 mRNA levels across all glioma grades^23^ using the Cancer Genome Atlas Project (TCGA) database. Public microarray database analyses revealed that CHD7 is up-regulated in tumor samples, when compared to normal brain tissue (NBT) (Fig. 1A), even though no significant alteration in genetic copy number was detected (see supplementary Fig. S1). Moreover, we found that CHD7 exhibited different expression patterns when comparing the four transcriptionally defined glioblastoma subtypes^24^ with higher levels in the proneural tumor samples (Fig. 1B).

Consistent with the TCGA interrogation, we confirmed increased CHD7 mRNA levels in glioma tissues by qRT-PCR (Fig. 1C). Next, we examined the presence of CHD7 expressing cells by immunohistochemistry in glioblastoma patient samples. We show that cells displaying high level of CHD7 protein are found within the tumor mass in the three different samples analyzed (Fig. 1D and supplementary Fig. S1). Altogether, these results show that CHD7 is up-regulated in at least a subset of gliomas irrespective of the grade.

### *CHD7* expression is highly heterogeneous in human glioblastoma-derived cell lines *in vitro*

**Figure 2:**
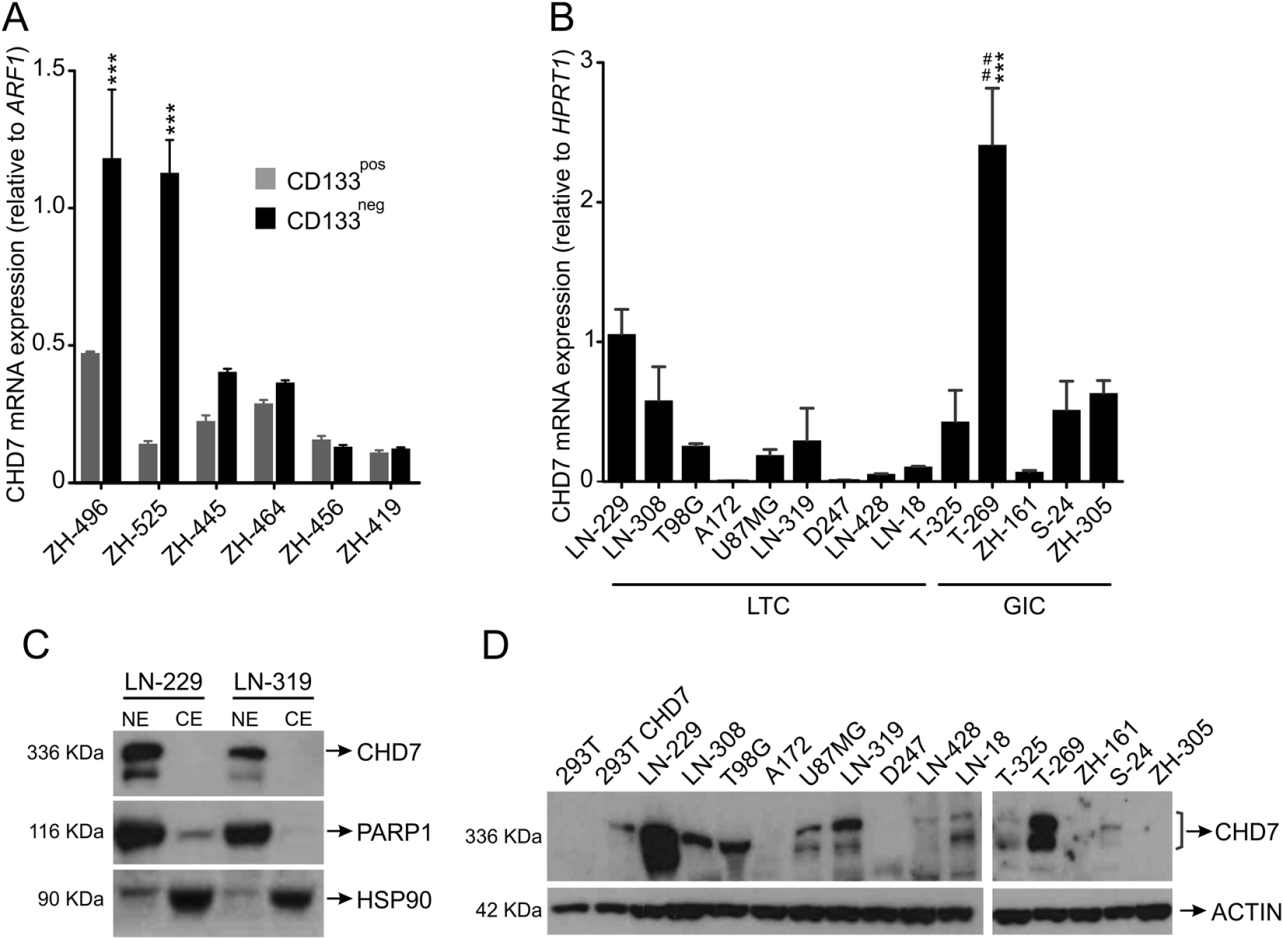
*CHD7* expression in freshly dissected tumor tissue and in human-glioblastoma derived cell lines. (**A**) Relative CHD7 mRNA levels of freshly dissociated CD133^pos^ and CD133^neg^ tumor cells were assessed by qRT-PCR. Cell fractions represent matched sub-populations from the same patient. Results are expressed as average ± SEM for technical replicates. ^***^p < 0.001; 2-way ANOVA followed by Bonferroni posttest. (**B**) Relative CHD7 mRNA levels in different LTCs and GICs, determined by qRT-PCR. The results represent average ± SEM from two independent experiments. ^##^p < 0.01, ^***^p < 0.001; one-way ANOVA followed by Bonferroni correction for multiple tests compared with LN-229 and all the other cell lines, respectively. (**C**) CHD7 immunoblotting of fractionated nuclear extracts (NE) and cytoplasmic extracts (CE) of LN-229 and LN-319 cell lines. PARP1 and HSP90 were used as nuclear and cytoplasmic markers, respectively, examined consecutively in the same blot as CHD7, after membrane stripping. (**D**) CHD7 immunoblotting of nuclear extracts of human glioblastoma-derived cell lines. Left panel shows the result from LTCs. In a separate gel, right panel, is the blot from the GICs. Actin was used as loading control for each of the gels. Due to the great difference in the protein sizes, the loading control was examined in a separate gel, loaded under the same conditions. Total protein extract of 293 T cells transfected with the empty vector and CHD7 overexpression plasmids were used as negative and positive controls, respectively.

To further characterize *CHD7* expression in glioblastoma, we used the CD133 cell surface marker to enrich for the glioblastoma-initiating cell (GIC) population^25^ from freshly dissected tumors. As measured by qRT-PCR, CHD7 mRNA levels were higher in CD133^neg^ sub-populations (Fig. 2A).

We next analyzed CHD7 mRNA and protein levels in a panel of eight human long-term glioblastoma cell lines (LTCs) and five GIC lines. We found that CHD7 is expressed in the vast majority of human glioblastoma-derived cell lines *in vitro* (Fig. 2B). In order to assess the CHD7 protein subcellular localization, we optimized immunoblotting with fractionated cytoplasmic and nuclear cell extracts, confirming CHD7 protein localization in the nucleus (Fig. 2C). Since CHD7 is mainly concentrated in the nucleus, we heretofore adopted blotting nuclear cell extracts instead of whole cell lysates in order to enhance CHD7 protein detection. Among the LTCs, the highest CHD7 protein levels were found in LN-229 cells, whereas T-269 cells displayed higher CHD7 protein levels among the GICs (Fig. 2D). Taken together, these results suggest that CHD7 is up-regulated in gliomas and its expression is highly heterogeneous in cultured glioblastoma cell lines.

### *CHD7* deletion attenuates anchorage-independent growth and spheroid invasion in LN-229 glioblastoma cell clones *in vitro*

Although *CHD7* expression was not significantly correlated to patient prognosis (see supplementary Fig. S2), the great heterogeneity within glioblastoma tumors prompted us to further examine the functional impact of CHD7 in glioblastoma cells endogenously expressing contrasting levels of this protein. Initially, we set out to investigate the effect of CHD7 loss-of-function in glioblastoma lines naturally expressing high levels of CHD7. For that purpose, we used the CRISPR/Cas9 genome editing technique to abrogate its expression in LN-229 cells.

**Figure 3:**
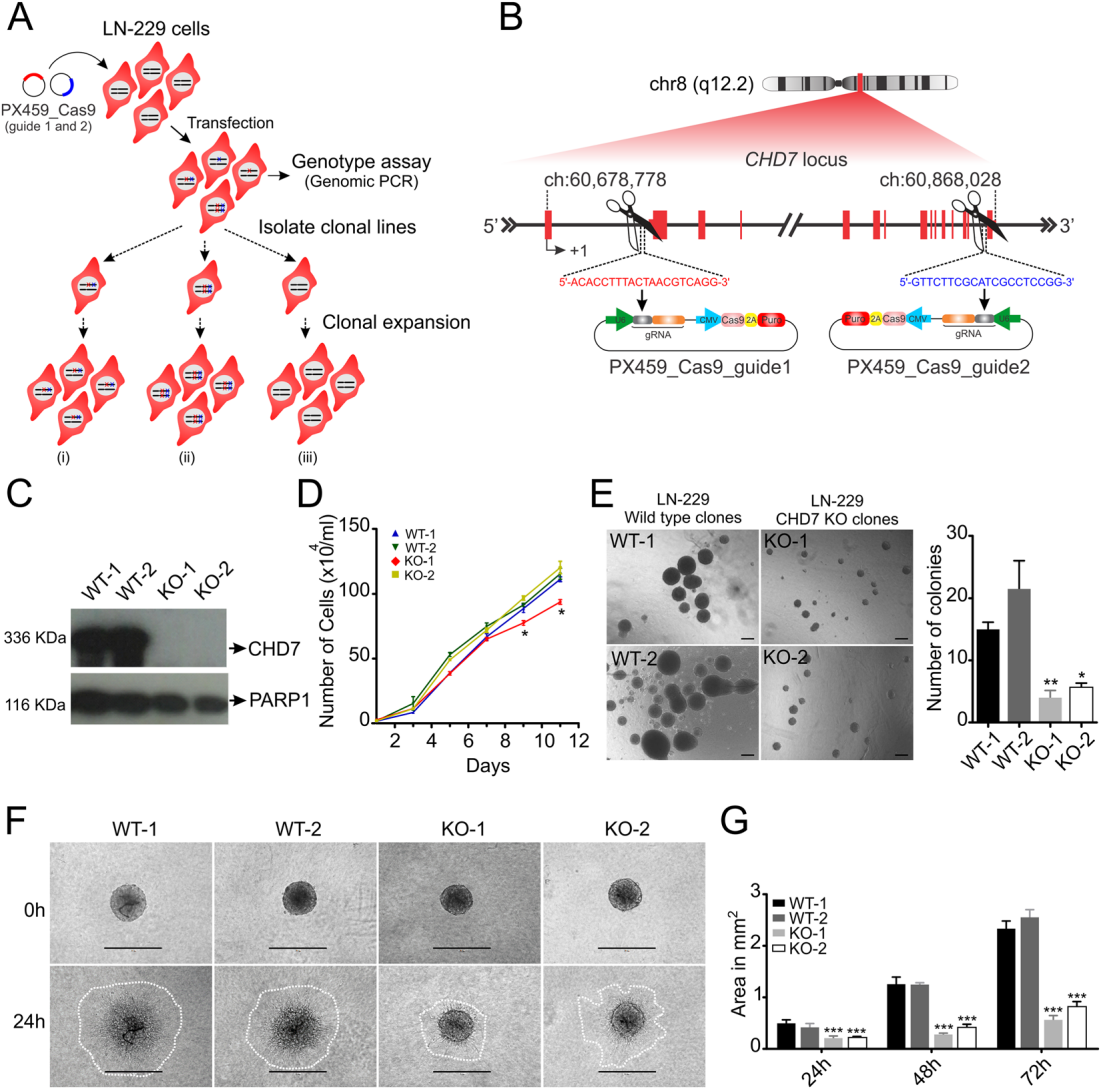
*CHD7* deletion inhibits anchorage-independent cell growth and spheroid invasion in LN-229 cells. (**A**) Strategy used to generate *CHD7* KO cell clones. LN-229 cell line was co-transfected with two sgRNAs and selected with puromycin for 48 h. After confirmation of genomic editing by PCR in the mixed population selected, we performed clonal isolation. (i-ii) indicate clones carrying *CHD7* mutations, which may lead to abrogation of *CHD7* expression, and (iii) indicates isolated clones which still exhibit *CHD7* expression. (**B**) Scheme indicating the sgRNA sequences targeting the 5′ and 3′ regions of the *CHD7* gene. (**C**) CHD7 immunoblotting of nuclear extracts from two WT and two KO isolated clones. PARP1 was used as the loading control, examined in the membrane as CHD7. (**D**) Growth curves of LN-229 clones. 1 × 10^4^ cells were plated in 12 well plates in triplicates for each time point. Experiments were performed three times. Results are expressed as average ± SEM for three wells of a single experiment. ^*^p < 0.05; one-way ANOVA followed by Dunnett’s test in comparison with WT-1. (**E**) 1 × 10^4^ LN-229 cells suspended in soft agar were layered onto the bottom agar in 24-well plates in triplicates. Representative images of cell colonies grown in culture medium for two weeks. Scale bar: 200 μm. The graph represents total number of colonies per well, greater than 50 μm diameter. Results are expressed as average ± SEM from three independent experiments. ^*^p < 0.05, ^**^p < 0.01; one-way ANOVA followed by Dunnett’s test in comparison with WT-1. (**F**) Spheroids of WT and KO clones were placed in a 3D-collagen I matrix and the area covered by invading cells was measured for quantification after 24, 48 and 72 h. Representative images show multicellular spheroids at the time point 0 h and 24 h. Scale bar: 400 µm. (**G**) Experiments were performed three times in quadruplicates for each cell clone. Results from a single representative experiment are presented. Results are expressed as average ± SEM. ^*^p < 0.05, ^**^p < 0.01, ^***^p < 0.001; 2-way ANOVA followed by Bonferroni test in comparison with WT-1.

Cells were transiently transfected with a combination of two different sgRNA/Cas9 constructs, targeting the initial and final region of the *CHD7* gene, aiming to delete most coding exons of *CHD7* gene or generate frame shift mutations. After confirming, by PCR, the deletion of genomic DNA in the cell population, we undertook single cell sorting in order to isolate cell clones (Fig. 3A,B). A total of 50 clones were expanded and PCR genotyped, showing that only a few clones presented the fragment deletion, while the amplification targeting the exon 3 sequence was also detected (Supplementary Fig. S3). Even though *CHD7* deletion did not seem to occur in both alleles, CHD7 immunoblotting showed that several samples did not express the CHD7 protein corresponding to the canonical transcript. Two independent cell clones, in which CHD7 protein was rendered undetectable (KO), along with two control clones, in which CHD7 expression was not altered (WT), were isolated for further analyses (Fig. 3C). All of these cell clones were successfully expanded in culture without any obvious loss in viability, although clone KO-1 displayed a slightly decreased, albeit significant, difference in growth rate (Fig. 3D).

Next, we analyzed anchorage-independent growth in a soft-agar colony formation assay. The total number of colonies greater than 50 µm diameter, was consistently decreased in the KO cell clones, when compared to the WT clones (Fig. 3E). Since anchorage-independent cell growth is associated with neoplastic transformation and metastatic potential, we asked whether the invasion capacity would be affected in the LN-229 KO clones. To that end, we used a 3D-collagen invasion assay to assess invasion in the LN-229 multicellular spheroids, at 24, 48 and 72 h culturing in a serum-containing collagen matrix (Fig. 3F). The area covered by the invading cells was reduced by about two-fold in the KO clones after 24 h, when compared to the *CHD7* expressing clones. The invaded area remained significantly reduced over time, indicating that the invasive potential of the cells was impaired upon *CHD7* deletion. Therefore, we demonstrated that CHD7 is not essential for LN-229 cell survival *in vitro*, however, its deletion affects their anchorage-independent growth and invasiveness.

### Ectopic *CHD7* overexpression elicits LN-428 cell migration and invasion *in vitro*

**Figure 4:**
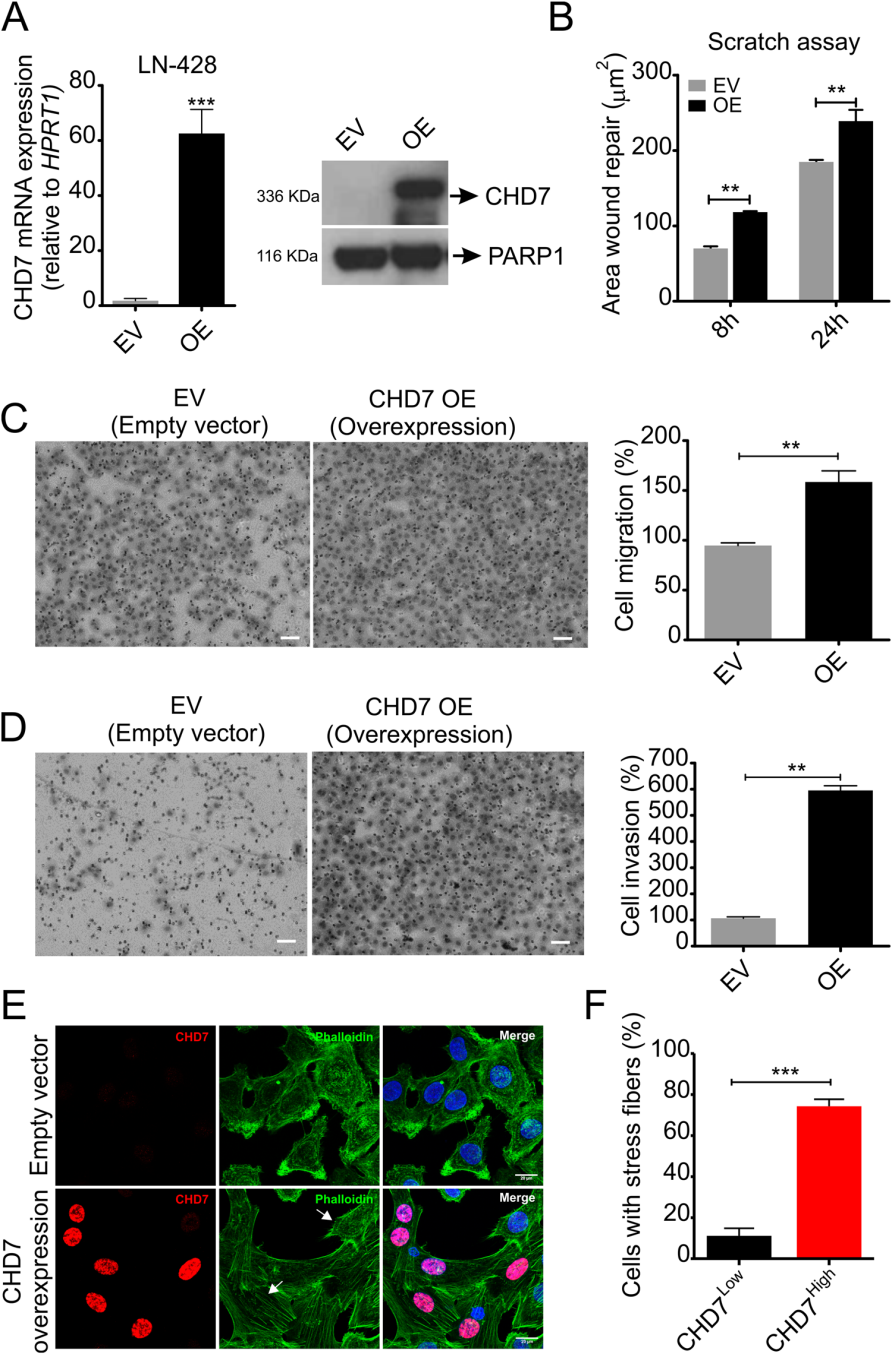
*CHD7* overexpression promotes migration and invasion of LN-428 cells *in vitro*. (**A**) LN-428 cells were transfected with either the EV or OE vectors and selected with 750 µg/mL G418. Relative CHD7 mRNA levels were assessed by qRT-PCR and immunoblotting of nuclear cell extracts. Results are expressed as average ± SEM. ^***^p < 0.001; Student’s t-test. PARP1 was used as loading control, examined in the same blot as CHD7. (**B**) Wound-healing scratch assay: 2 × 10^5^ cells were plated in 24 well plates in triplicates. Experiments were performed three times. Results are expressed as average ± SEM for three wells of a single experiment. ^**^p < 0.01; 2-way ANOVA. (**C**) Representative images and statistical plots of transwell migration assays. (**D**) Representative images and statistical plots of transwell Matrigel coated invasion assays. Scale bar: 100 µm. The number of cells which transversed the membrane was assessed after 16 h incubation and six independent fields at 10x magnification objective were counted for each well. Three independent experiments using duplicates were performed for each assay. Results are expressed as average ± SEM. ^**^p < 0.01; Student’s t-test. (**E**) *CHD7* overexpression promotes changes in the cytoskeleton of LN-428 cells. The upper panel shows LN-428 EV and the lower panel shows LN-428 OE. White arrows indicate differential cytoskeleton organization in a cell that does not display high CHD7 protein level, in comparison with OE cells. Images were captured using confocal microscope. CHD7 (red), Actin filaments (green) and nuclei (blue). Scale bar: 20 µm. (**F**) Graph shows the percentage of LN-428 cells with low and high CHD7 protein levels which display evident stress fibers. EV and OE cells were plated in three different passages and four independent fields at 20x magnification objective were counted for each well. ^***^p < 0.001; Student’s t-test.

To determine whether the reduced cell invasion capacity observed in LN-229 KO cell clones might originate from a direct effect of *CHD7* deletion, we selected the LN-428 cell line, which expresses low level of endogenous CHD7 protein, to generate a cell population that constitutively overexpresses CHD7 (OE). To this end, the full-length *CHD7* cDNA was cloned into the mammalian pCXN2-DEST expression vector harboring a very strong promoter for mammalian cells (see supplementary Fig. S4). LN-428 cells were transfected with the empty vector (EV) or the CHD7-expressing construct and the G418-resistant polyclonal cell populations were selected and expanded for further analysis. Quantification by qRT-PCR and immunoblotting confirmed CHD7 overexpression in the LN-428 cell line (Fig. 4A).

We first performed a scratch wound healing assay and we observed that OE cells possessed approximately 30% increased migration potential compared to EV (Fig. 4B). We also found that *CHD7* ectopic expression increased, by almost two-fold, the transwell migration capacity of LN-428 cells (Fig. 4C). We further investigated the role of CHD7 in modulating tumor cell invasion using the Matrigel invasion assay. CHD7 significantly enhanced, by about six-fold, the invasion capacity of LN-428 cells across the transwell chamber, when compared to cells transfected with the EV (Fig. 4D).

Immunofluorescence staining of actin filaments was carried out to evaluate whether cytoskeletal alterations could be associated with altered cell motility capacity. We observed over 40% increase in the number of cells displaying stress fibers in OE cells when compared to cells which do not express high levels of the protein (Fig. 4E,F and supplementary Fig. S5).

Similarly, A172 OE cells display significant changes in cell motility and invasiveness, although in a lesser extend (see supplementary Fig. S6). Together, these data strongly indicate that CHD7 plays an important role in glioblastoma cell migration and invasion.

### CHD7 modulates tumor growth in orthotopic xenograft mouse glioma models

**Figure 5:**
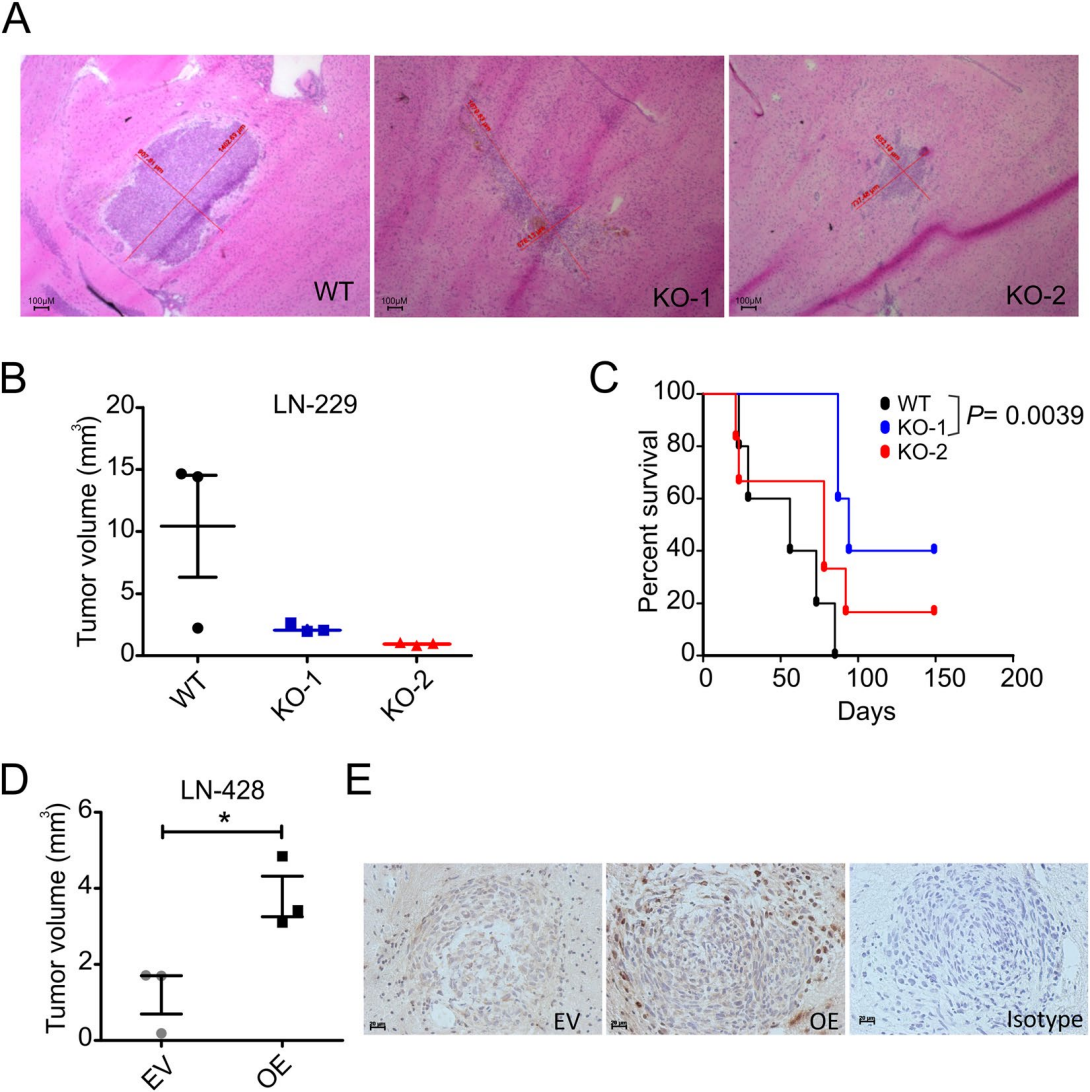
Effect of altered *CHD7* expression in tumor development *in vivo*. (**A**) 7,5 × 10^4^ human LN-229 cells were orthotopically implanted into the right striatum of nude mice. For histological analysis, three animals per group were sacrificed on the same day when the first animal(s) became symptomatic. Brain sections were examined and tumor sizes were assessed on H&E-stained sections. Scale bar: 100 µm. (**B**) Each tumor is indicated by a dot. The bar marks the average ± SEM (n = 3, p > 0.05; one-way ANOVA compared with WT clone followed by Bonferroni correction for multiple tests). (**C**) Kaplan–Meier survival curves (n = 5; p < 0.05; Gehan-Breslow-Wilcoxon test). Animals were maintained until the onset of clinical grade 2 symptoms. (**D**) Three animals per group were inoculated with 1 × 10^5^ LN-428 cells stably transfected with EV and OE vectors. For histological analysis, animals were sacrificed on the same day when the first animal(s) became symptomatic. LN-428 tumor size was assessed on H&E-stained sections. Each tumor is indicated by a dot. The bar marks the mean ± SEM (n = 3, ^*^p < 0.05; Student’s t-test). (**E**) Representative CHD7 immunohistochemistry in LN-428 EV and in LN-428 OE tumors. Scale bar: 20 µm.

To investigate whether CHD7 is relevant for tumor development and progression, we analyzed whether perturbation of CHD7 protein levels affects the tumorigenic potential of glioblastoma cells in an orthotopic xenograft murine glioma model. Athymic nude mice were used for stereotactical implantation of cells derived from one LN-229 WT clone and two KO clones (n = 8). To measure tumor volume, three pre-randomized mice of each group were sacrificed on the day when the first animal developed neurological symptoms. Analysis of brain sections showed that none of the animals injected with KO cell clones developed big tumors at the time point analyzed, suggesting a delay in tumor growth progression (Fig. 5A,B). Likewise, mice inoculated with the KO-1 clone experienced prolonged survival rate compared to the WT clone, whereas animals injected with clone KO-2 cells showed a similar effect, although the differences were not statistically significant when compared to the WT clone (Fig. 5C).

In a similar setting, animals were inoculated with LN-428 OE and EV and the tumor volume was measured (n = 3). The tumor size was significantly increased in OE group, when compared to EV (Fig. 5D). Immunohistochemistry of brain sections showed that cells displaying high protein levels are located at the border of the tumor (Fig. 5E) suggesting a correlation between the levels of ectopic *CHD7* overexpression and the migration and invasion phenotypes of LN-428 cells *in vivo*.

Altogether, our results demonstrate that functional deletion of CHD7 in human glioblastoma cells that express high levels of CHD7, may lead to decreased tumor progression, whereas ectopic overexpression of this protein in human glioblastoma cells which express low levels of CHD7, enhances tumor growth and increases cell invasiveness *in vivo*.

### Modulation in CHD7 levels altered the expression of adhesion molecules

Due to the apparent association of CHD7 with glioblastoma pathogenesis, as well as the previously described function of CHD7 in gene transcription^10,11,13^, we set out to perform gene expression profiling aimed at gathering molecular insights of the role of CHD7 in human glioblastoma. For that purpose, we carried out whole transcriptome/RNA-seq analysis of those same engineered cell lines in which CHD7 expression has been perturbed.

The comparison between three distinct WT and three isolated *CHD7* KO LN-229 cell clones, reveled 307 differentially expressed genes (DEGs) whose expression was significantly altered (FDR < 0.05; abs(LFC) > 1, case over control; see supplementary Fig. S7 and supplementary Table S4). In LN-428 cells, we identified 869 DEGs in OE when compared to EV transfected cells (see supplementary Fig. S7 and supplementary Table S5).

Notably, 58 transcripts were commonly regulated in both cell lines, whereas 18 presented alterations in opposite directions in the expression levels between these two groups (Table 1).

**Table 1:** Commonly regulated DEGs between LN-229 KO × WT and LN-428 OE × EV.

Symbol	Name	LN-229 KO	LN-428 OE
**CHD7**	**Chromodomain Helicase DNA Binding Protein 7**	**−1**.**8**	**2**.**8**
**AIF1L**	**Allograft Inflammatory Factor 1 Like**	**−1**.**8**	**1**.**5**
**ZNF502**	**Zinc Finger Protein 502**	**−5**.**0**	**1**.**0**
C4orf26	Odontogenesis Associated Phosphoprotein	4.8	−1.2
FRMPD4	FERM And PDZ Domain Containing 4	1.2	−1.2
MSC-AS1	MSC Antisense RNA 1	2.4	−1.2
ABCA1	ATP Binding Cassette Subfamily A Member 1	1.0	−1.3
CCL5	C-C Motif Chemokine Ligand 5	3.3	−1.3
KIF26B	Kinesin Family Member 26B	1.5	−1.4
PXYLP1	2-Phosphoxylose Phosphatase 1	1.0	−1.6
SLCO1A2	Solute Carrier Organic Anion Transporter Family Member 1A2	1.2	−1.6
CHMP1B2P	Charged Multivesicular Body Protein 1B2, Pseudogene	2.0	−1.7
MYH16	Myosin Heavy Chain 16 Pseudogene	1.9	−2.1
RN7SL2	RNA, 7SL, Cytoplasmic 2	1.4	−2.1
RN7SL3	RNA, 7SL, Cytoplasmic 3	1.2	−2.2
HAPLN1	Hyaluronan And Proteoglycan Link Protein 1	2.7	−2.7
BMPR1B	Bone Morphogenetic Protein Receptor Type 1B	1.2	−3.0
ZNF618	Zinc Finger Protein 618	2.8	−3.1

The list presents the DEGs which showed expression level changes in opposite directions, with fold change (FC) values expressed as log_2_ scale over control. Negative and positive values indicate down-regulation and up-regulation of gene expression, respectively. Genes are listed in descending order of FC values in OE. Bold DEGs indicate genes with decreased expression in KO and increased expression in OE, followed by the other genes which showed increase expression in KO and decreased expression in OE. Significant changes were considered for FDR < 0.05; abs (LFC) > 1, case over control.

**Figure 6:**
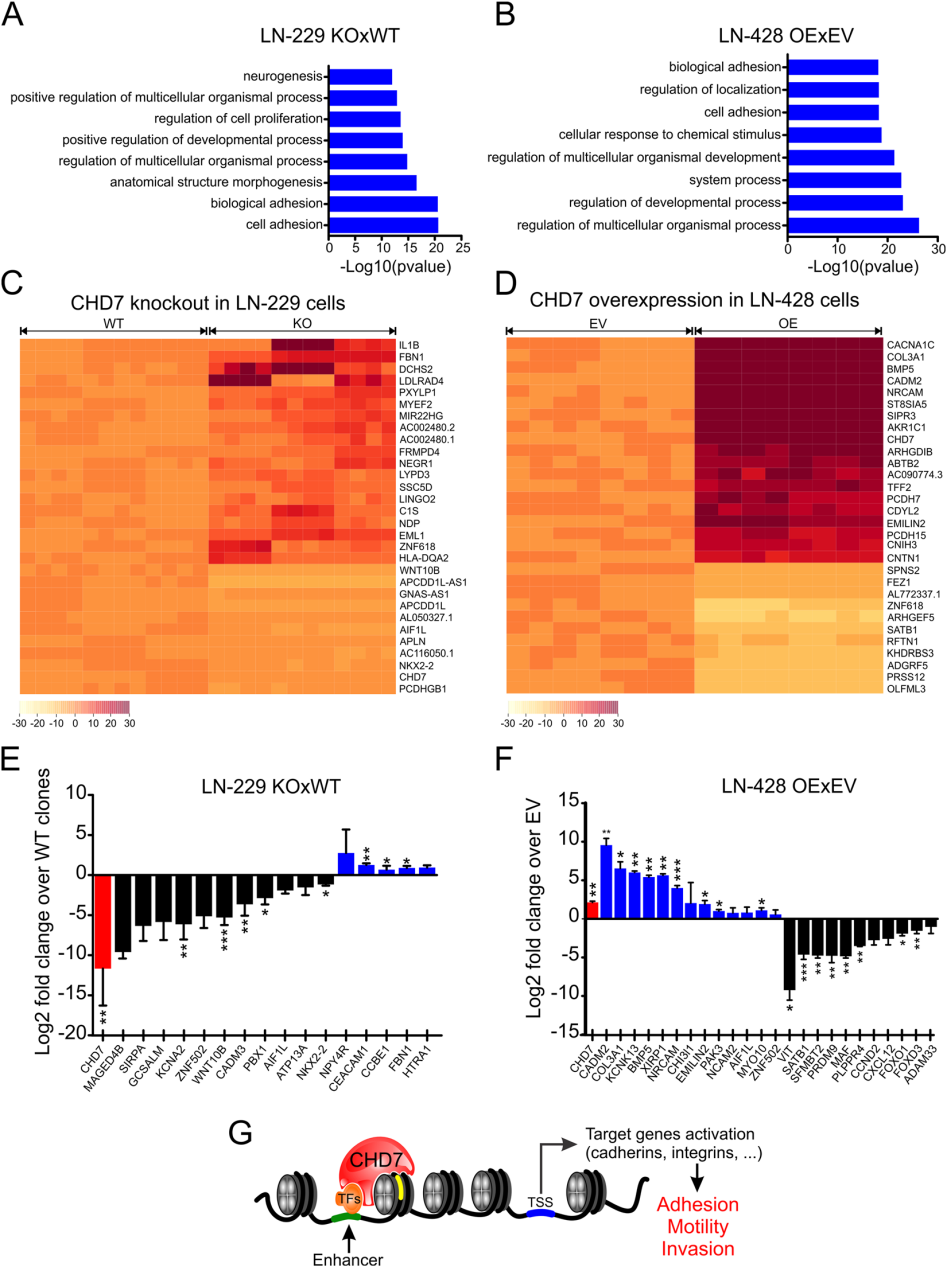
Differential transcriptome analysis of LN-229 KO cell clones and LN-428 OE cell population. (**A** and **B**) Gene ontology analysis indicating the most enriched pathways for the significantly altered genes in the modified cell lines, defined by STRING database. (**C** and **D**) Heat-maps indicating the shortlisted genes which are most significantly modulated under both comparative conditions. (**E** and **F**) Validation of gene expression by qRT-PCR. *CHD7* is indicated in red. Down-regulated (black) and up-regulated (blue) genes related to tumorigenesis, cell migration or cell invasion. Values are the means ± SEM (n = 3, ^*^p < 0.05, ^**^p < 0.01, ^***^p < 0.001; Student’s t-test). (**G**) Proposed model for CHD7 regulation of glioblastoma cell motility and invasiveness. CHD7 is recruited to binding sites through interactions with cell type-specific transcription factors (TFs) and histone modifications. The energy provided by ATP hydrolysis enables nucleosome translocation revealing extra TF-binding sites (yellow rectangle). Binding of additional TFs, associated with co-activators, promotes DNA accessibility favoring transcription. In glioblastoma cells, CHD7 modulates the expression of several adhesion molecules, such as integrins and cadherins, stimulating cell motility and invasiveness.

Even though CHD7 seems to regulate distinct genes in LN-229 and LN-428 cells, the altered genes were highly associated with pathways such as “biological adhesion”, “cell adhesion” and “locomotion” in gene ontology (GO) analysis (Fig. 6A,B and supplementary Fig. S7).

Genes encoding proteins involved in cell-cell adhesion, including several integrins (*ITGA1*, *ITGBL1*, *ITGB2*, *ITGB3*) and protochaderins/cadherins (*PCDH7*, *PCDH10*, *PCDH15*, *CDH7*, *CDH9* and *CDH15*), as well as other cell binding molecules, such as CADM2, CADM3, NRCAM, NRCAM2, EMILIN2 and CNTN1, were also modulated upon changes in *CHD7* expression.

The heat-maps indicate the top 30 DEGs in both groups (Fig. 6C,D). To independently validate these results, changes in the expression of 40 genes associated with tumorigenesis, cell motility or invasiveness were analyzed by qRT-PCR (Fig. 6E,F).

Next, we sought to compare the differentially expressed genes found in our model with previously described CHD7 localization determined by chromatin immunoprecipitation in NSCs^13^. These authors revealed approximately 16,000 binding sites near or in the gene sequences. We found that 28% (85 genes) of the DEGs in KO and 30% (259 genes) of the genes modulated in OE coincide with CHD7 binding sites previously mapped in mouse NSCs (see supplementary Table S6). These data suggest that CHD7 might have binding sites and/or transcriptional targets that are conserved in between mouse NSCs and human glioblastoma cells.

## Discussion

CHD7 is known to be essential for organogenesis^26^ and in the non-pathological brain, it was shown to be crucial for NSC function^13–15,27^. On the other hand, the involvement of CHD7 in tumorigenesis has just begun to be described, gaining considerable attention in the last few years.

*In silico* analysis of 32 tumor types revealed that *CHD7* is the most commonly gained/amplified and mutated gene among the CHD members. The same study also showed that overexpression of *CHD7* was more prevalent in aggressive subtypes of breast cancer, being significantly correlated with high tumor grade and poor prognosis^16^. Consistent with these findings, frequent *CHD7* mutations have been reported in stomach and colon cancers^19^ and aberrations in CHD7 activity was suggested to contribute to colorectal carcinoma CpG island methylator phenotype 1^18^. Moreover, low *CHD7* expression in the G4 meduloblastoma subtype, in combination with *BMI1* overexpression, has recently been shown to contribute to tumor formation^17^. Mechanistically, CHD7 inactivation favors chromatin accessibility at BMI1 target genes, which, in turn, leads to ERK over-activity and increased cell proliferation. Thus, these data strongly suggest the association of *CHD7* with the pathology of different human cancers.

In the present study, we provide evidence that CHD7 plays an important role in glioblastoma pathogenesis, which is the most common and deadliest of malignant primary brain tumors in adults^28^. Despite the multimodality treatment, which typically includes surgery, ionizing radiation, and cytotoxic chemotherapy, the average overall survival rate remains at only ∼15 months, highlighting the urgent need for more effective targeted therapeutics^29^.

Our results indicate that *CHD7* is highly expressed in glioma patient samples, as previously suggested^30^. Additionally, using the CD133 cell surface marker, we observed that *CHD7* is more expressed in the CD133 negative cell population in a subgroup of tumor samples. We also demonstrated that *CHD7* knockout not only inhibited anchorage-independent growth in LN-229 cells, but also reduced cell invasion ability. Interestingly, CD133 immunostaining in glioma patient samples showed that CD133 expression was significantly reduced in migrating tumor cells in the tumor periphery compared to tumor cells in the core region^31^. However, the same study discusses that CD133 was found at lower level compared to other stem cell markers, such as Nestin, Musashi-1 and SOX-2.

*In vitro*, *CHD7* expression was found to be highly heterogeneous in the panel of human glioblastoma-derived cell lines analyzed consistent to what we observed in our set of clinical samples as well as in the TCGA public database. Importantly, the cell line presenting the highest CHD7 protein level was the T-269 GIC. Ohta and colleagues have also shown that *CHD7* is found to be highly expressed in different established GICs, when compared to normal human astrocytes^30^, however, what specifically determines the great variability in *CHD7* expression among these cell lines remains elusive but a warranted field of future investigation.

Studies on CHD7 domain-specific functions and overexpression phenotypes are still scarce in the literature, possibly due to the length of the coding region (2,997 aa) and technical difficulties in generating expression vectors^32^. A recent study showed that introduction of a mRNA encoding the CHD7 isoform 2 (948 aa), in KhES-1 human embryonic stem cells, induced spontaneous cell differentiation *in vitro* and *CHD7*-overexpressing cell culture could not be maintained^33^. Here, we amplified the *CHD7* coding sequence as three overlapping fragments and the full-length was then assembled and inserted into the expression vector by Gateway®-assisted sub-cloning. We demonstrated that ectopic *CHD7* expression enhanced migration and invasion capacity of LN-428 cells, potentially by regulating stress fiber assembly and adhesion dynamics. Actin stress fiber formation is one of the critical steps associated with cell invasion^34^, but the precise mechanism by which CHD7 promotes reorganization of the cell cytoskeleton remains to be elucidated. We were unable to explore the role of CHD7 in GICs using the same approach, due to difficulties in selecting and expanding modified cells for experiments.

Interestingly, *CHD7* duplications have been suggested to be a driver mutation identified in small-cell lung cancer, one of the most highly metastatic and aggressive types of cancer^21^. Additionally, using iPS-derived neural crest cells from CHARGE patients, *CHD7* mutations were also found to promote defective cell migration^35^. This study showed modulation of several genes related to cell adhesion and migration, such as *CTGF*, *COL3A1*, *SERPINE1* and *THBS1*, all of which we found to be modulated in glioblastoma cells. *CHD7* has also been implicated in regulation of neural crest cell migration during embryogenesis in *Xenopus*^7^. In this model and also in human neural crest cells, CHD7 was shown to associate with PBAF to modulate *SOX9* and *TWIST1* gene expression, which are essential for proper cell migration of this cell type. We did not observe significant changes in those genes in our model, indicating that CHD7 may regulate cell motility by different mechanisms, possibly by association with cell-specific interaction partners.

In fact, it has been previously shown that CHD7 binding sites present high variability among different cell types and CHD7 binding itself is context-dependent (e.g., embryonic cell in the differentiated state showed only 30% overlap in the binding sites)^10^. In our study, 18 genes were commonly regulated in opposite directions in LN-229 and LN-428 cell lines. Moreover, the comparison with previously described CHD7 binding sites reveled that genes modulated by CHD7 perturbation in glioblastoma cells, such as NRCAM, CNTN1 and EMILIN2, have significantly higher enrichment of CHD7 occupancy in mouse NSCs^13^, suggesting direct transcription modulation of these targets. Importantly, several of these genes have been implicated in glioblastoma invasiveness^36^.

One could argue that each cell type in a given tissue might have unique CHD7 binding sites and protein complexes, which vary over time^32^. Our findings demonstrate the diversity of CHD7-regulated genes and suggest a broader function for CHD7 as a master regulator of cell migration and invasion. It will be of great interest to investigate, for example, the underlying mechanism regulating differential *CHD7* expression in glioblastoma cells and whether these pathways are amenable to manipulation by molecular intervention aiming at clinical therapeutic trials.

## Conclusions

The invasive behavior of malignant gliomas is one of the most important characteristics which contributes to tumor recurrence after surgery^37^. Our data provides functional and molecular evidences for a novel oncogenic role of CHD7 as a transcriptional regulator of pro-invasive and motility factors in glioblastoma cells (Fig. 6G). Further studies may warrant important clinical-translational implications for glioblastoma treatment.

## Methods

### Gene expression and survival analysis using The Cancer Genome Atlas (TCGA) dataset

Overall expression analysis within the TCGA database (http://cancergenome.nih.gov) was undertaken using the single gene expression analysis module of the R2: microarray analysis and visualization platform (http://www.r2.amc.nl). CHD7 expression clusters were generated across 276 samples (MAS5.0 - u133p2 dataset) and analyzed with k-means algorithm and log_2_ transformation of gene expression. Analysis of CHD7 expression relative to the glioblastoma subtype^24^ was carried out using 435 samples classified into these groups within the subtype track mode and z-score transformation. Kaplan-Meier survival analysis and log-rank tests were carried out as detailed in Supplementary Information.

### Patient samples

Brain tissue samples from temporal lobectomy epileptic patients and from resected astrocytoma specimens were macro-dissected and immediately snap-frozen in liquid nitrogen^38^. The specimens were categorized according to the 2007 WHO classification^23^. This project has the approval of the Ethical Committee of the University of São Paulo School of Medicine (CAPPesq, 691/05), and informed consent was obtained from all patients. The CD133^pos^ and CD133^neg^ cells were isolated from freshly resected human glioblastoma tumor tissue (ZH-419, ZH-445, ZH-456, ZH-464, ZH-496 and ZH-525) after written informed consent of the patients and approval by the Institutional Review Board of the University Hospital Zurich. Detailed protocol described in Supplementary Information.

### *In vitro* assays

Details on cell lines, reagents, real-time quantitative reverse transcription-PCR (qRT-PCR) and primers, are provided in Information. Detailed protocol for immunofluorescence, anchorage-independent clonal growth, migration and invasion assays is summarized in Supplementary Information.

### CRISPR/Cas9 knockout of CHD7

CHD7 knockout clones were generated according to the protocol described by Ran and colleagues^39^. Briefly, small guide RNAs (sgRNAs) were designed, using an CRISPR Design Tool (http://tools.genome-engineering.org) and then cloned (guide sequences in Supplementary Material and Methods Table S2) into the pSpCas9(BB)-2A-Puro (PX459), a gift from Feng Zhang (Addgene plasmid #48139, Addgene, Massachusetts, USA). Lipofectamine 2000 (Life Technologies) was used for transient co-transfection of two sgRNAs constructs at a 1:1 ratio. Cells were selected with 3 μg/mL puromycin (Life Technologies) for 48 h and genomic DNA was extracted using QIAamp DNA Kit (Qiagen, Venlo, Netherlands) for detection of the CHD7 deletion by PCR (primer sequences available in Supplementary Information Table S3). Transfected cells were then isolated by BD FACSARIA II (BD Bioscience) single cell sorting in 96 well plates. Cell clones were expanded for genomic DNA extraction and genotyping (Supplementary Fig. S3). Selected clones were further expanded for nuclear protein extraction and tested by immunoblotting.

### CDH7 overexpression

A 9 Kb cDNA, comprising the ORF full-length of the human *CHD7* gene (GenBank Accession #NM_017780.3) was amplified by long RT-PCR from the OVCAR8 human ovarian cancer cell line as three overlapping ~3 kb fragments. Briefly, total RNA was purified from OVCAR8 cells (RNeasy RNA Purification Kit, Qiagen) and 1 μg RNA was used as the template for reverse transcription with SuperScriptIII® (Life Technologies). PCRs were carried out using Phusion^®^ High Fidelity DNA Polymerase (New England Biolabs, Ipswich, MA) and the resulting PCR products were cloned using TOPO^®^ Zero Blunt cloning kit (Thermofisher). Clones displaying the correct sequence, as judged by Sanger sequencing, were assembled by Gibson^®^ cloning into the full length ORF using the unique *AflII* and *MfeI* restriction sites of the CHD7 cDNA. A Kozak consensus sequence was added juxtaposed to the initial ATG codon for optimal expression levels in mammalian cells. The final full-length 9Kb CHD7 cDNA was cloned into the pCXN2 expression vector^40^ using the Gateway^®^-assisted sub-cloning.

To generate OE cell populations, cells were transfected with the pCXN2_CHD7 construct or with the empty vector using Lipofectamine 2000 (Life Technologies). LN-428 and A172 transfected cells were selected with 750 µg/mL and 200 µg/mL of Geneticin G418 Sulfate (Gibco, Thermo Scientific), respectively.

### Histology and immunohistochemistry

Three glioblastoma patient samples (ZH149, ZH265, ZH276) and one normal brain tissue were used to investigate CHD7 protein levels. Samples were de-paraffinized and rehydrated tumor tissue sections were boiled in EDTA buffer, pre-treated with 1% H_2_O_2_ and blocked in blocking solution (Candor Biosciences, Germany). Sections were incubated with primary anti-CHD7 antibodies (ab31824, 1/200) (Abcam, Cambridge, UK) at 4 °C overnight. Simultaneously and under the same conditions, matching rabbit IgG isotype control (ab27478, 1/200) was used in place of the CHD7 primary antibody for accurate interpretation of immunostaining results. After washing, samples were incubated with goat anti-rabbit IgG-AP (sc2007, 1/200) (Santa Cruz, Texas, USA) at room temperature for 30 min (under protection from light). The DAB+ (#K3468, Dako) chromogen substrate was used as the detection system and the sections were counterstained with Mayer’s hematoxylin to visualize the nuclei.

Tumor-bearing brains were embedded in cryomoulds in Shandon Cytochrome yellow (Thermo Scientific, Waltham, MA) and frozen in liquid nitrogen. Tumor incidences and sizes were determined using (H&E)-stained 8 μm thick cryosections using a Microm HM560 (Microchom HM560, Thermo Scientific).

### Immunoblotting

Total cellular extracts were obtained by lysing cells with RIPA buffer (150 mM, NaCl, 1% NP-40, 0.5% SDS, 50 mM Tris pH 8.2, 1 mM EDTA). Cytoplasm and nuclear protein lysates were prepared with the NE-PER Nuclear and Cytoplasmic Extraction kit (Thermo Scientific). Proteins (30 µg per lane) were resolved on a 3 to 7% Tris-acetate gel (Life Technologies) to detect CHD7 and PARP1. Actin was evaluated in a 10% SDS-PAGE. Gels were transferred to a nitrocellulose membrane (Life Technologies). After blocking with 0.5% non-fat milk in TBS containing 0.5% Tween 20 (TBST), the membrane was incubated in blocking solution with primary antibody overnight at 4 °C. After washing and incubation with the HRP-conjugated secondary antibody (1/5,000, Sigma Aldrich), the protein bands were detected with enhanced chemoluminescence (ECL, Thermo Scientific). Original blots are presented in Supplementary Information, Fig. S8.

### RNA-seq experiment and data analysis

The next-generation sequencing (NGS) libraries were prepared according to Illumina TruSeq Stranded mRNA LT protocol. Quality control of the amplified products before and after fragmentation and labeling was analyzed using the Agilent Bioanalyzer. Samples were sequenced on Illumina NextSeq. 550 (2 × 76 bp paired-end sequencing) operated by the Biomedical Institute Facility Center CEFAP of the University of São Paulo (USP). All calculations were carried out as described in Supplementary Information.

### Animal studies

All experiments were carried out according to the Swiss Federal Law on the Protection of Animals, the Swiss Federal Ordinance on the Protection of Animals, and the guidelines of the Swiss confederation (permission #ZH062/15). FoxN1 nu/nu mice (Charles River, Sulzfeld, Germany) aged between 6–12 weeks were anaesthetized and placed in a stereotaxic fixation device. A burr hole was drilled in the skull 2 mm lateral and 1 mm posterior to the bregma. The needle of a Hamilton syringe was introduced into a depth of 3 mm^41^. LN-229 (7.5 × 10^4^) and LN-428 (1 × 10^5^) cells were resuspended in PBSA and then injected into the right striatum. Animals were clinically assessed three times per week and sacrificed upon developing neurological symptoms, justifying euthanasia (score 2).

### Statistics

Analysis of the relative mRNA levels between different glioma grades and glioblastoma samples were carried out by a non-parametric analysis of variance (Kruskal-Wallis test) with Dunn test for post-hoc comparison. *In vitro* experiments were performed in biological and technical replicates. Results are expressed as the mean and SEM of triplicate determinations. The statistical analyses were performed by unpaired Student’s t-test or ANOVA for multiple comparison tests. Animal survival statistics was assessed using Gehan-Breslow-Wilcoxon test. All statistical analyses were carried out using Prism 5 (GraphPad Software, La Jolla, CA).

### Ethics approval and consent to participate

Clinical human samples were obtained from patients undergoing surgical resection. All the procedures were performed in accordance with the guidelines and regulations as determined by the Ethical Committee of the University of São Paulo School of Medicine (CAPPesq, 691/05), and informed consents were obtained from all patients. The CD133^pos^ and CD133^neg^ cells were isolated from freshly resected human glioblastoma tumor tissue after written informed consent of the patients and in accordance with the guidelines and regulations determined by the Institutional Review Board of the University Hospital Zurich. All animal experiments were performed in accordance with protocols approved by the Swiss Federal Law on the Protection of Animals, the Swiss Federal Ordinance on the Protection of Animals, and the guidelines of the Swiss confederation (permission #ZH062/15).

## Supplementary information

Supplementary Information

Supplementary Table S4

Supplementary Table S5

Supplementary Table S6

## Data Availability

The dataset of RNA-seq has been deposited at NCBI Gene Expression Omnibus (http://www.ncbi.nlm.nih.gov/geo/). Accession number will be release when the manuscript is approved.
