# Supplementary material for: CHD7 promotes glioblastoma cell motility and invasiveness through transcriptional modulation of an invasion signature
Source: Sci Rep. 2019 Mar 8;9:3952. doi: 10.1038/s41598-019-39564-w (PMC6408455; doi:10.1038/s41598-019-39564-w)
Supplement: Supplementary file 1 — Supplementary Information [file 41598_2019_39564_MOESM1_ESM.docx]

Supplementary Information

**CHD7 promotes glioblastoma cell motility and invasiveness through transcriptional modulation of an invasion signature.**

Raquel A. C. Machado, Hannah Schneider, Carlos DeOcesano-Pereira, Flavio Lichtenstein, Fernando Andrade, André Fujita, Marina Trombetta-Lima, Michael Weller, Christian Bowman-Colin, Mari Cleide Sogayar

**Supplementary Material and Methods**

**Gene expression and survival analysis using The Cancer Genome Atlas (TCGA) dataset**

Kaplan-Meier survival curves and log-rank tests were constructed to evaluate differences in overall survival of predicted good or poor prognosis groups determined by CHD7 gene expression. Patients were classified into the good and poor prognosis groups (low and high risk, respectively) using the median cutoff of the CHD7 expression value.

To better evaluate the association between CHD7 gene expression and survival, we used a Cox regression model to minimize the influence of clinical variables. Patient data such as age at diagnosis, gender, chemotherapy, and tumor subtype were included as covariates. We only considered individuals presenting all information regarding these covariates (i.e., 446 out of 558 patients). For survival probability, gene expression data were normalized to zero mean and unit variance. All computations were carried out in the R statistical environment (https://www.r-project.org/). For Cox regressions we used the R package survival.

**CD133^pos^ and CD133^neg^ cell populations**

Tumor tissues were dissociated with 10 mg/mL collagenase/dispase (Roche, Basel, Switzerland) and gentle rotation in MACS C-tubes (Miltenyi biotech, Cologne, Germany). Discrimination of cell populations was achieved using MicroBeads conjugated to the mouse anti-human CD133/1 epitope antibody (clone AC133) (Miltenyi Biotec), followed by depletion of CD45+ cells utilizing MACS LS columns (Miltenyi Biotec). Cells were immediately lysed after magnetic sorting and total RNA for qRT-PCR was prepared using the NucleoSpin System (Macherey-Nagel) and complementary DNA transcribed using SuperScript® II reverse transcriptase (Invitrogen). Differential CD133 expression was confirmed by analyzing CD133 mRNA levels in both cell populations, using Arf1 transcript levels as a house-keeping reference for relative quantification (primer sequences in Table S1).

**Cell lines and reagents**

The human A172, U87MG and T98G long-term cell lines (LTCs) were purchased from the American Type Culture Collection. The LN-18, LN-229, LN-308, LN-319 and LN-428 cell lines were kindly provided by Dr. N. De Tribolet (Lausanne, Switzerland). LTCs were maintained in Dulbecco’s modified Eagle’s medium (DMEM, Invitrogen, Life Technologies, Carlsbad, CA), containing 10% fetal calf serum (FCS) (VWR Lonza, Leighton Buzzard, UK) and supplemented with 2 mM glutamine (Invitrogen, Life Technologies), in a 5% CO_2_ incubator at 37°C. The S-24, T-269, T-325, ZH-161 and ZH-305 primary patient-derived glioma-initiating cells (GICs) were generated and cultured as described ^1,2^. Briefly, GICs were maintained as sphere cultures in Neurobasal A medium (Invitrogen, Life Technologies) supplemented with EGF (10 ng/mL), FGF (10 ng/mL) (Peprotech, Rocky Hill, NJ), heparin (31.5 U/mL) (Sigma Aldrich, St. Louis, MO), 1% Glutamax (Invitrogen, Life Technologies) and 2% B27 (Invitrogen, Life Technologies).

**Quantitative Real time PCR (qRT-PCR)**

For mRNA expression analysis, total RNA was transcribed into cDNA using the iScript cDNA Synthesis Kit (Bio-Rad Laboratories, Hercules, CA). cDNA amplification was monitored using SYBR Green chemistry and the ViiA™ 7 Real-Time PCR System (Applied Biosystems, Foster City, CA). Conditions for PCR reactions were: 40 cycles of 95°C/15 sec, 60°C/1 min, using the specific primers listed in this file, Table S1.

**Antibodies used for immunoblot**

Primary antibodies: anti-β-actin (csc-1616, 1/2,000) (Santa Cruz Biotechnology, Dallas, TX), anti-­CHD7 (ASB453, 1/500) (Millipore, Billerica HQ, MA), anti-LaminB1 (Ab 16048, 1/2,000) (Abcam, Cambridge, UK), anti-PARP (556494, 1/2,000) (BD Biosciences, Franklin Lakes, NJ).

**Immunofluorescence**

For immunofluorescence, cells were washed with PHEM buffer (2 mM HEPES, 10 mM EGTA, 2 mM MgCl_2_, 60 mM PIPES – pH 6.9) and fixed for 30 min with cold PFA 4%. Cells were permeabilized with 0.1% Triton X-100 for 10 min, blocked with 3% goat serum (DY005, R&D Systems, Minneapolis, US) for 1h, and then incubated with anti-CHD7 (ab 176807, 1:1000) (Abcam) overnight at 4°C. After PHEM washing (3x), the cells were incubated with AlexaFluor 594 goat anti-rabbit IgG (A11012, 1/1000) and Alexa fluor 488-Phalloidin (A12379, 1/1,000) (Thermo Fisher Scientific) at room temperature for 1h. Coverslips were mounted using VECTASHIELD Anti-fade Mounting Medium with DAPI (H-1200, Vector Laboratories, CA) and images were acquired with a confocal Zeiss LSM 780-NLO microscope.

To quantify cells displaying stress fibers, cells from LN-428 EV and OE in three consecutive passages were plated at low density in P12 well plates on top of coverslips. After two days, coverslips were fixed and all samples were stained simultaneously as described above.

**Anchorage-independent clonal growth in semi-solid medium**

Anchorage-independent clonal growth was assessed using the soft-agar assay ^3^. Briefly, 10^4^ cells/well were seeded on top of the 0.6% agarose (Fisher Scientific, Leicestershire, UK) solution in 10% FCS-DMEM in a 24 well plate. Cells were plated onto the 0.6% agarose layer and allowed to stand for about 10 min before the addition of 500 μL of melted 0.3% agarose in 10% FCS-DMEM. Finally, 500 μL of liquid 10% FCS-DMEM were added. This liquid medium was renewed every two days and cells were allowed to grow for about 14 days, forming large colonies, which were then quantified using the AMG EVOS FL Inverted Microscope.

**Migration and Invasion assays**

For the Transwell assay, cells were washed in phosphate buffered saline (PBSA) and ressuspended in serum-free medium. A cell suspension containing 50,000 cells was added to the upper well of transwell migration inserts (pore size: 8 μm, BD Biosciences) or to BD BioCoatTM MatrigelTM invasion chambers (pore size: 8 μm, BD Biosciences). In the lower well, 700 μL of complete medium were used as chemo-attractant. The cells were maintained for 16h at 37°C and 5% CO2, followed by fixation in cold methanol for 10min and staining with Mayer’s alum haematoxylin for 20 min. Inserts were mounted in glass slides and six fields per sample were counted, with duplicates for each treatment.

For wound-healing scratch assay, 2x10^5^ LN-428 cells were plated in 24 well plates in triplicates. On the following day, the cell layers were scratched using a 200 μl sterile pipette tip. The wound location was marked and images of the same field were captured to record the wound width at 0, 8 and 24 h.

For the spheroid invasion assays, LN-229 and A172 spheroids were generated by incubating 1,000 cells for 72h in 96-well plates pre-coated with 1% Noble Agar (Difco Laboratories, Detroit, MI). Spheroids with a diameter of 200 µm were embedded into a collagen matrix containing collagen type I (Invitrogen), 10% FCS and 10% NaHCO3 in a 96-well plate. Sprouting of spheroids was monitored daily by photographs. For quantitation, the area covered by sprouting cells was assessed using the ImageJ 1.40g software (NIH). For measurement of the invasion area, the area covered by the spheroid at Day 0 was subtracted from the overall area, which was covered on subsequent days.

**RNA-seq experiment and data analysis**

All calculations were carried out using Linux Scripts and R-Studio IDE (R language). To map and quantify the transcripts, we used the Subread ^4^ aligner package. Samples were then normalized and the Differentially Expressed Genes (DEGs) were calculated using EdgeR ^5^ package. The mean transcription counts were determined through the biological coefficient of variation, using a negative binomial distribution, resulting in counts per million (CPM) estimation. DEG between Overexpressed (case) and Empty Vector (control) samples; and Knockout (case) and Wild Type (control) samples were calculated. DEGs are defined as genes with absolute value of log2 fold change (LFC) > 1, p-value < .05 and false discovery rate (FDR) < .05 between case and control. The levels of gene expression of selected targets were validated by qRT-PCR. Differential gene set analysis was carried out using the Panther Classification System and String-db.

**Table S1: Primer sequences used in qRT-PCR.**

| Gene | Forward (5' to 3') | Reverse (5' to 3') |
| --- | --- | --- |
| Arf1 | GACCACGATCCTCTACAAGC | TCCCACACAGTGAAGCTGATG |
| CD133 | TGGATGCAGAACTTGACAACGT | ATACCTGCTACGACAGTCGTGGT |
| HPRT1 | TGAGGATTTGGAAAGGGTGT | GAGCACACAGAGGGCTACAA |
| CHD7 | CAGAACACCCCGCAGAAAGTGCCTGT | AGCATTCGGTCCACTAACCTGAGTCAT |
| CADM2 | CCCTCCCTTACCACTGCAA | GCCAGCCAAAGCATTAGGA |
| RIMS1 | CAGCCGAGCCGAGAGTCTA | CAGCCGAGCCGAGAGTCTA |
| COL3A | AAAACCCCGCTAGAAACTGC | GCATCCAATTTGCATCCTTG |
| KCNK13 | CTTCCTCCGCCACTACGAG | TCCTACTGTCGCCGGAGTT |
| BMP5 | TGTGCAGAAACAGGGGATG | TTTGTTGGCTGCTCTCACG |
| XIRP1 | GTCAGTGCAACTCGCTGGA | GATGCTGCTGCTGCTGAAC |
| CHI3L1 | TTCCGAGGTCAGGAGGATG | CACCAGCTTACTGGCAGGAG |
| CNTN1 | AGCAACCCTGAGCTTTGGA | GGGGGTCACAGAGAAGCAC |
| PAK3 | ATCGCACCAAGACCAGAGC | TTCAGCAGAGGGTGGTGTG |
| NCAM2 | TCGTATGTGATGCGGAAGG | ATGCTGCCCTTTGACTTCG |
| AIF1L | GAGGTGACAGGAGGGGTCA | TGCTCTCGTTGGCTTTTCC |
| ADAM33 | CTGGCCTGGTGTTGCTACC | CCAACTCCATGGGGTGAAC |
| MYO10 | TATGGCTCGACGCTGTTTG | CTTCCCTCTCCACGCTTGT |
| ZNF502 | TCTGCATCAGTGGGAAACAA | TGGGTAAGGGATGAGCTCTG |
| SIRPA | CATCCACAGCACAGCCAAG | TCCAAGGTGGGTGGAACTC |
| MAGED4B | CAACAGCAACCCACCTGAG | TTCTGATTCTGGGCGATGA |
| WNT10B | CTGGTGAGCTGTGGCTGTG | TTGTGGATTCGCATTCGTG |
| KCNA2 | GTGGGGTGACCTTCCACAC | GCTGGGACAGGCAAAGAAC |
| GCSAML | TCCTCCCTGAGCTCCAATG | ATCATGCTCATGGGTGCAG |
| CADM3 | TACCTCTACGCCCCACGAG | GCAGTTCGCACAGGCATAG |
| ATP1A3 | GCCTTCCAGAATGCCTACC | TCACAGTCGAAGGCAAAGC |
| NKX2-2 | CGAGGGCCTTCAGTACTCC | GTCATTGTCCGGTGACTCG |
| VIT | CATGGCTGCCCATCTGAAG | GGGGTGAGTGGCAATGACT |
| PLPPR4 | GCGGGCTAACACGGATAAC | TTCCCCACAGCATACAAGC |
| SATB1 | GTGACAGATGCCCCTGATG | GTTCGGGAGGCAAGTCTTC |
| SFMBT2 | CCCCAGAGAGGACACGAAG | TCCTCCTCCTCCTGTTTCG |
| PRDM9 | CCAGTGTCCCCTTCTGGAG | GGCTCGCTGACCTCTTTGT |
| MAF | ATCCGGCTGAAGCAGAAGA | TGAGGTGGTCGACTTGCTG |
| FOXO1 | AGCGTGCCCTACTTCAAGG | TGGATTGAGCATCCACCAA |
| FOXD3 | TGCGAGTTCATCAGCAACC | GGGGATCTTGACGAAGCAG |
| CCND2 | TGTTCCTGGCCTCCAAACT | GTTCCCACTCCAGCAGCTC |
| CXCL12 | ATGCCGATTCTTCGAAAGC | TGTTCTTCAGCCGGGCTAC |
| NPY4R | TCCCACTGGGCTTCATCTT | CACCAGCACCACATTGACC |
| CCBE1 | CTCTGCTCCCCAACAATGC | TCCCTTTGGTCCTGGTGAG |
| FBN1 | CCTTACCTGGCGGAAATCA | CTGGAGCCACAGGAAGGAG |
| HTRA1 | ACGGTGCCACTTACGAAGC | GATGGCGACCACGAACTCT |
| PBX1 | CAGCAACCCTTACCCCAGT | CCGGATTCGCTTATTTCCA |
| EMILIN2 | CTGTGCCTGGAACCAGATG | AGGACAGCACCTCCATTCC |
| NRCAM | CTCCAGAAGGCAATGCAAG | TGGGTAGCATTCCATCTTCC |
| CEACAM1 | CGTCACCCAGAATGACACAG | TCCTCCACAGGGTTGGAGT |

**Table S2: Primer sequences used to clone the sgRNAs targeting CHD7.**

| Guide | Forward (5' to 3') | Reverse (5' to 3') |
| --- | --- | --- |
| sgCHD7-5' | CACCGACACCCTTACTAACGTCAGG | AAACCCTGACGTTAGTAAGGGTGTC |
| sgCHD7-3' | CACCGGTTCTTCGCATCGCCTCCGG | AAACCCGGAGGCGATGCGAAGAACC |

**Table S3: Primer sequences used in genomic PCR for LN-229 cell clones genotyping.**

| Primer | 5' to 3' | Fragment size |
| --- | --- | --- |
| Control_Fw | ATGCAGGAGAGATGTGAGTCTTAGAGATTTAACTGA | 729 bp |
| Control_RV | GTATTTATGTTGCTTTCTTATGTGGTTTGGGGGTAG |  |
| Exon3_Fw | GAAGAAGGGCTGGAATGCTTAGTGAAATATAGTGAC | 680 bp |
| Exon3_Rv | AGAGGATCCCAGTAAAGGTTTTGGTAAAGATGACTT |  |
| Deletion_Fw | GAGTTTTTGAATAGGAGCACCACCGCTTAACGTCAC | ~ 870 bp |
| Deletion_Rv | ACTGCACAATACTTAATGACCAAGATACCTTTTGAC |  |

**Supplementary Figures**

Figure S1


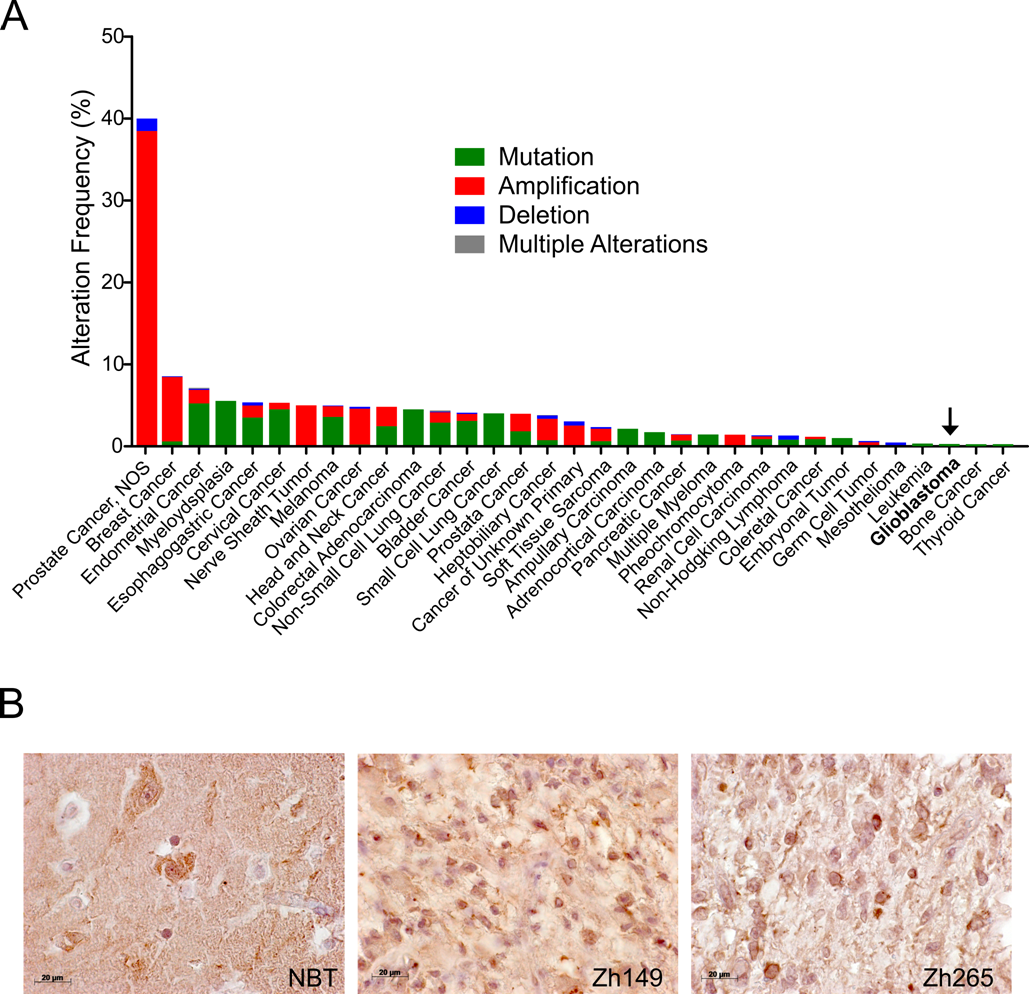


**Figure S1: *CHD7* mutations are rare in glioblastoma and cells expressing high levels of the protein are presente in glioblastoma patient samples.** **(A)** Frequencies of *CHD7* genetic alterations across 33 human cancers. Mutations in *CHD7* were found in 0.29% (7 cases) of 2,454 glioblastoma samples. The TCGA database was analyzed via cBioportal. **(B)** Representative images of CHD7 immunohistochemistry in normal brain tissue (NBT) and in ZH149 and ZH265 glioblastoma patient samples. Scale bar = 20 μm.

Figure S2


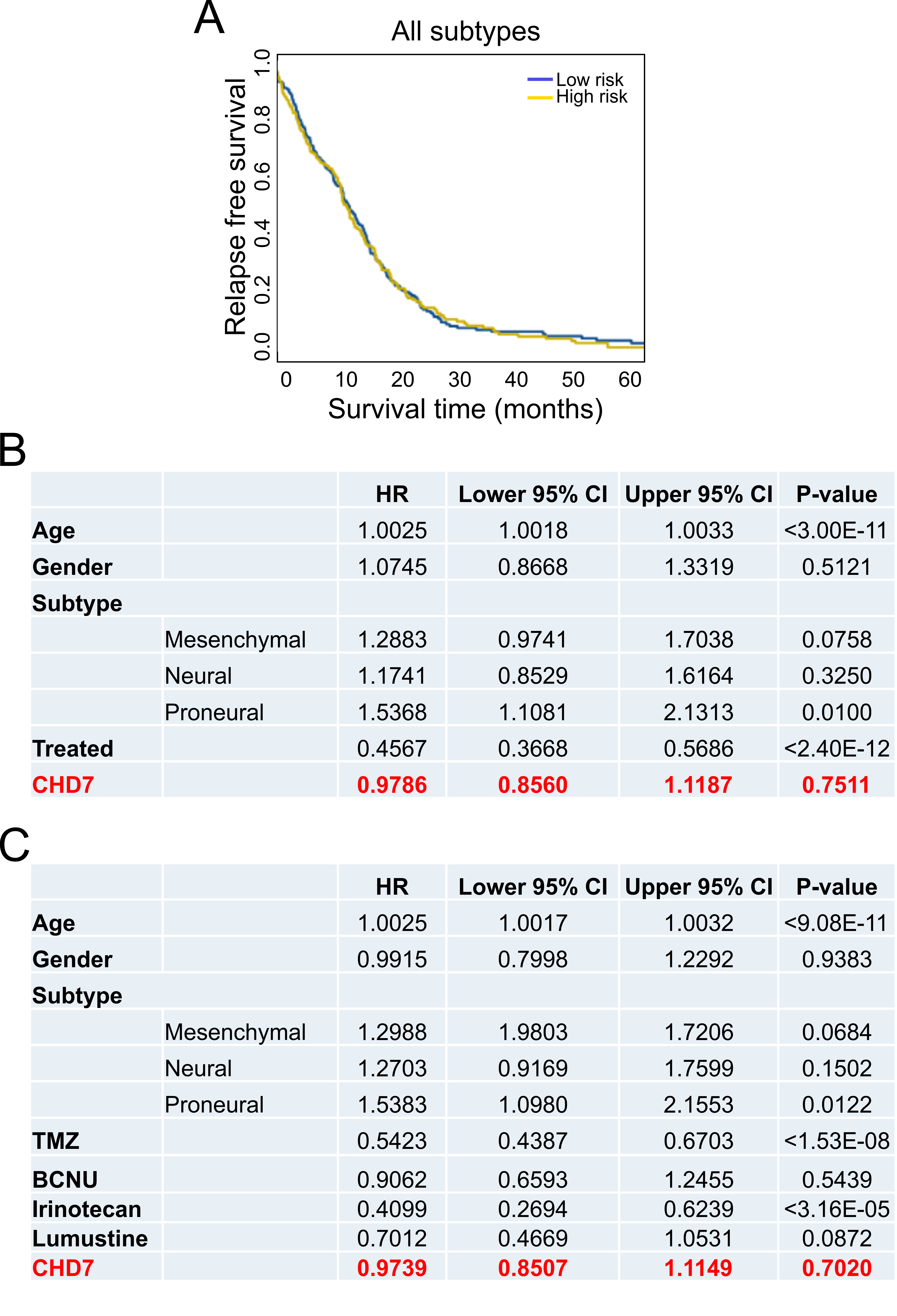


**Figure S2****: CHD7 expression is not directly correlated to patient survival.** **(A)** Kaplan-Meier analysis of CHD7^high^ and CHD7^low^ in glioblastoma patients from TCGA. Analysis for all glioblastoma samples (p= 0.77). **(B)** Cox regression analysis. "Subtype" contrasts individuals from different glioblastoma subtypes to individuals classified as "Classical". "Treatment" contrasts individuals treated with any chemotherapy drug from those without chemotherapy. **(C)** Cox regression analysis. Subtype contrasts individuals from different glioblastoma subtypes to individuals classified as "Classical". Temozolomide (TMZ), carmustine (BCNU), irinotecan and lomustine are dichotomous variables, indicating whether the patient was treated (or not) with that drug. CHD7 gene expression, on log2 scale, is normalized to zero mean and unity variance. HR - Hazard Ratio, CI - Confidence Interval.

Figure S3


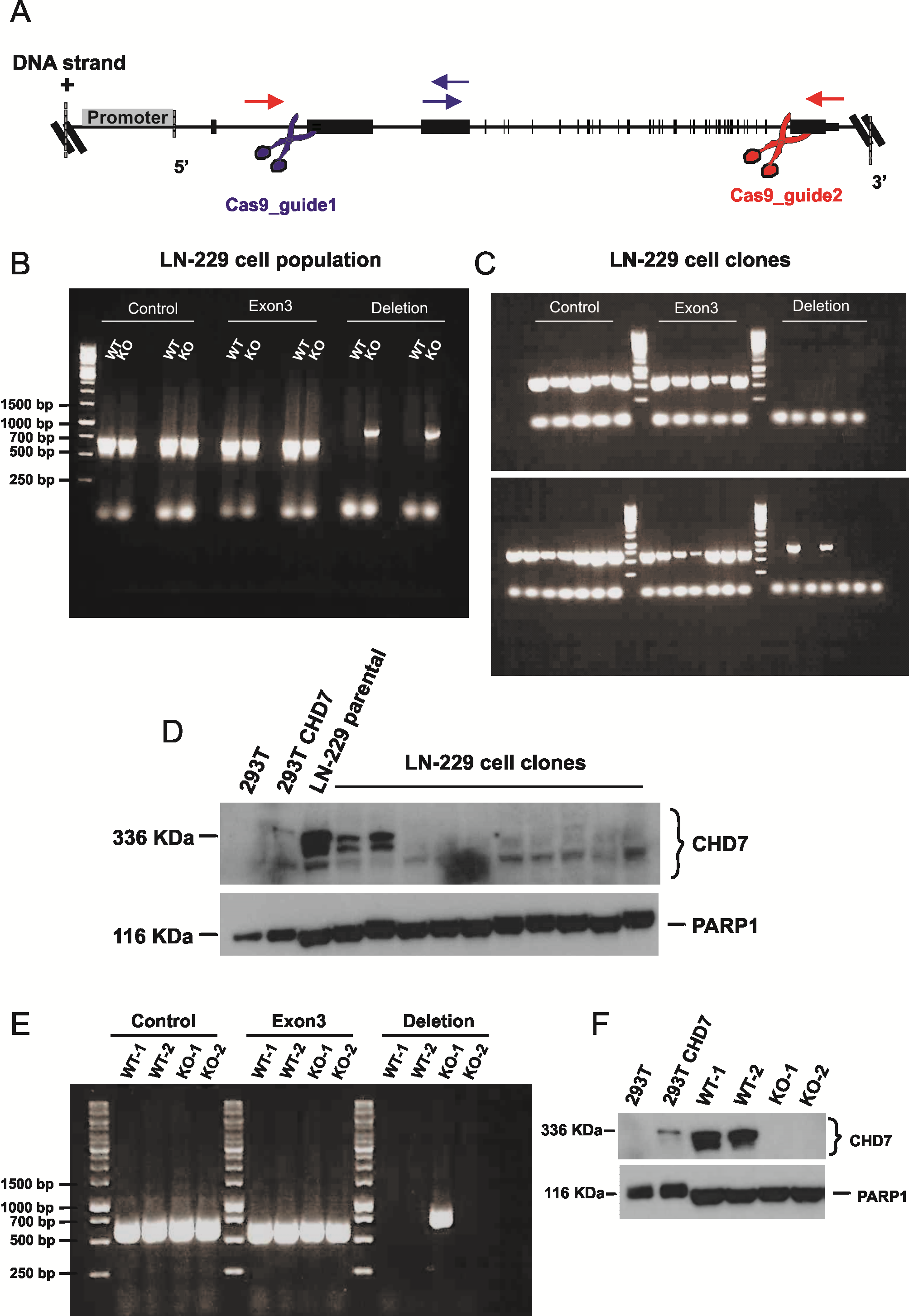


**Figure S3: LN-229 cell clones genotyping. (A)** Strategy used to detect *CHD7* deletion. A pair of primers designed to amplify a fragment not related to the target region was used as control (729 bp). Blue arrows represent primers designed to detect the presence of exon 3 (680 bp). Red arrows represent the primers designed upstream and downstream of sgRNAs target sequences (~870bp) to detect complete deletion. **(B)** PCR results carried out after LN-229 cell transfection with the combination of sgRNAs. LN-229 non-transfected cells (WT) were used as negative control. Experiment was performed in duplicate. Amplification of the fragment in the transfected cell population indicates that deletion has occurred. **(C)** Example of PCR results carried out in the LN-229 cell clones. 11 out of 50 cell clones were positive for *CHD7* deletion; however, exon 3 amplification suggests that total deletion in both alleles did not occur. **(D)** Example of CHD7 immunobloting carried out to characterize WT and KO cell clones. **(E)** and **(F)** Characterization of the cell clones used in the functional studies. KO-1 displays *CHD7* deletion whereas exon 3 is still present. KO-2 does not display CHD7 deletion. CHD7 protein was consistently not detectable in both KO cell clones.

Figure S4


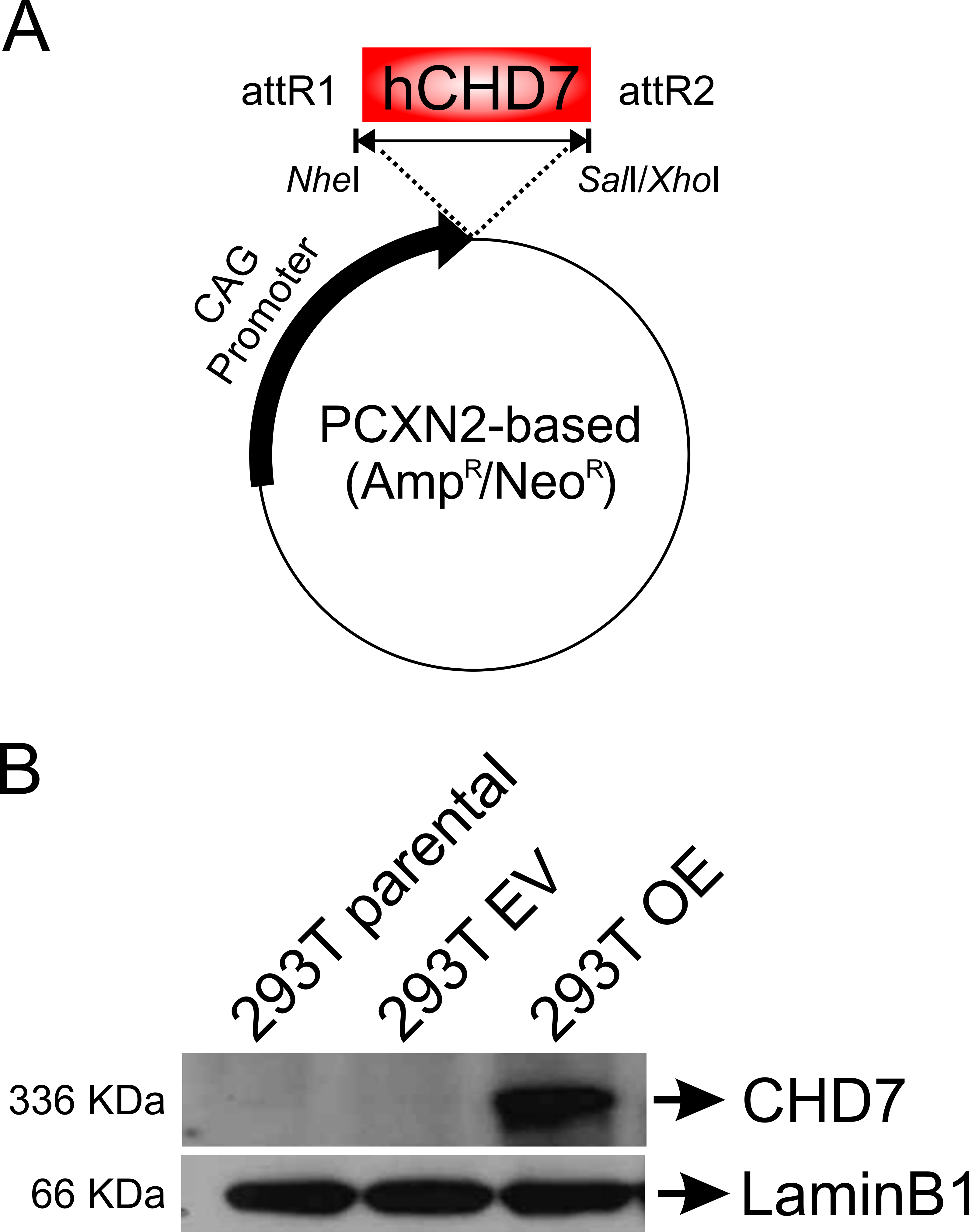


**Figure S4: CHD7 overexpression construct.** (**A)** A full-length CHD7 cDNA was built and inserted into a Gateway(R)-adapted pCXN2 mammalian expression vector ^6^. **(B)** Plasmids were transfected into 293T cells and protein expression was assessed by immunoblotting of total protein cell extracts. LaminB1 was used as loading control.

Figure S5


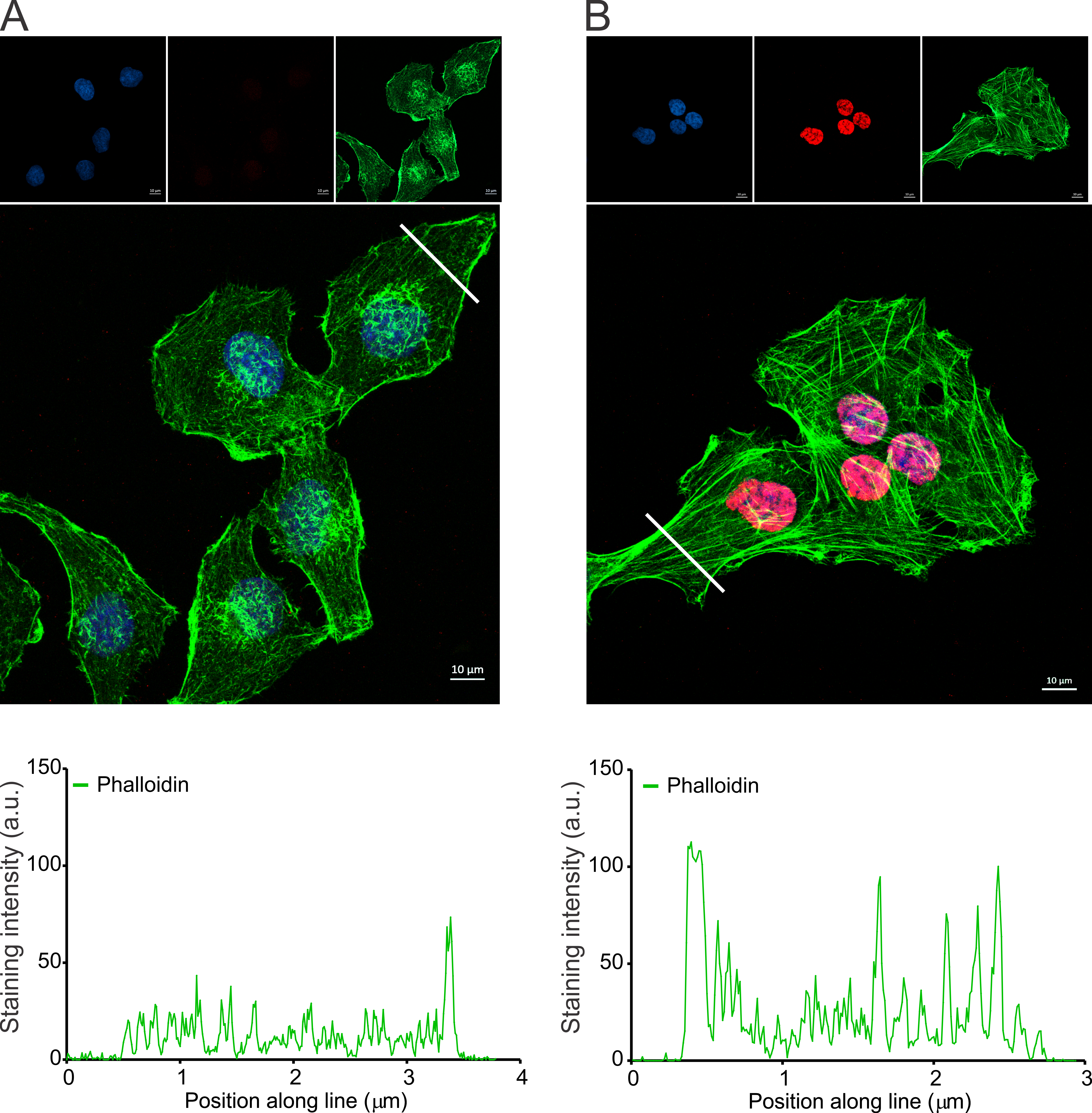


**Figure S5: Actin filament reorganization in LN-428 CHD7 overexpressing cells.** Representative maximum projection images of confocal Z-stacks showing LN-428 cells. Red: CHD7, Green: Phalloidin, Blue: DAPI. Scale bar: 10 µm. Graphs show line scans along the white line used to analyze the distribution of F-actin (ImageJ, plot profile function). Lines were drawn perpendicular to stress fibers across the cell cytoplasm. The normalized intensity profiles are shown. **A)** Representative image and intensity of F-actin staning in LN-428 with low CHD7 levels. **B)** Representative image and intensity of F-actin staning in LN-428 with high CHD7 levels.

Figure S6


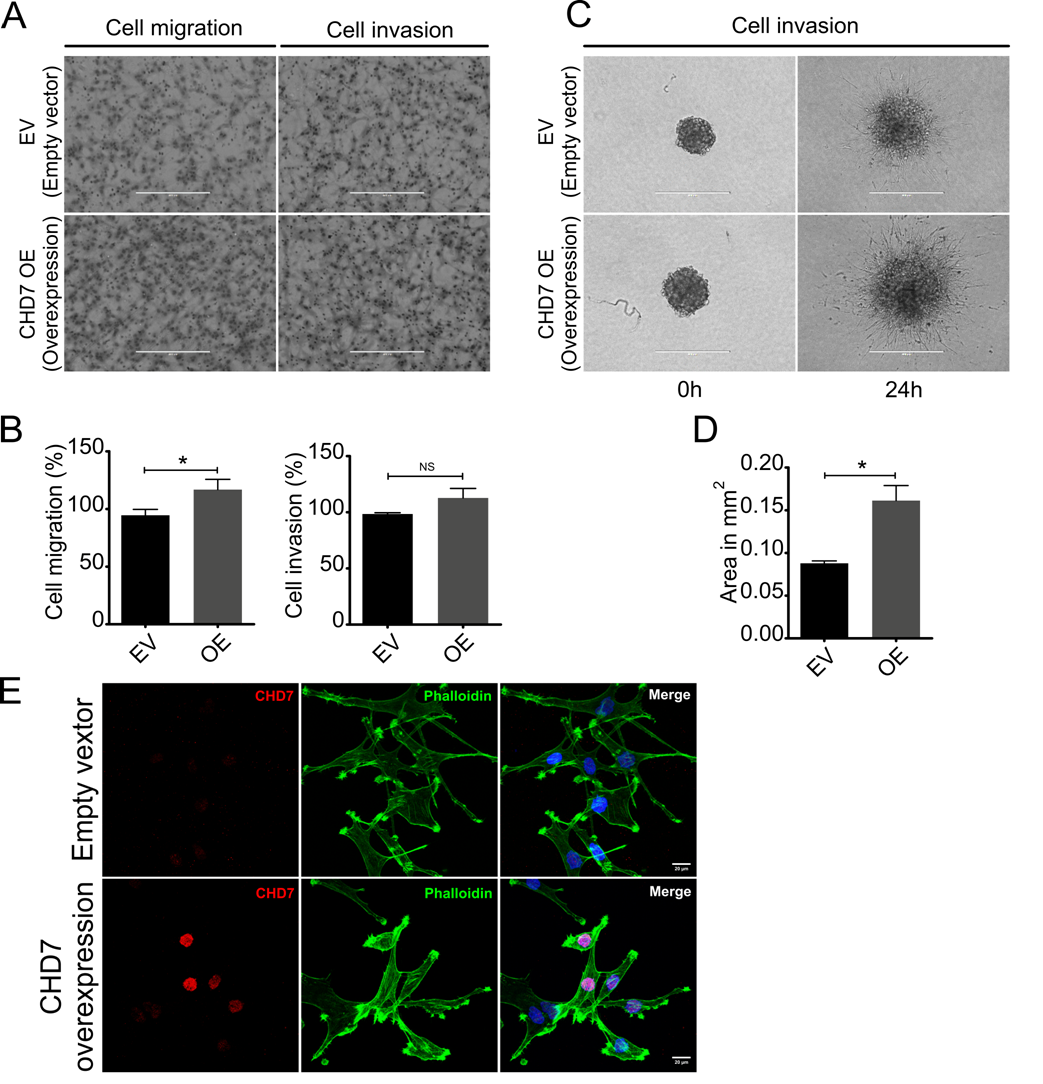


**Figure S6: CHD7 modulates A172 cell motility and invasiveness.** **(A)** and **(B)** Representative images and statistical plots of transwell migration (left painel) and transwell Matrigel coated invasion assays (right panel). The number of cells which transversed the membrane was assessed after 16h incubation and six fields were counted for each well using a 10x magnification objective. Three independent experiments using duplicates were performed for each assay. **p<0.01; Student’s t-test. **(C)** Multicellular spheroids of EV and OE clones were placed in a 3D collagen I matrix and the area covered by invading cells was quantified after 24h. **(D)** Experiments were performed three times in triplicates. Results from a single representative experiment are presented. Values are expressed as average ± SEM. *p<0.05; Student’s t-test. Scale bar: 400 µm. **e** Immunofluorescence of A172 EV and OE cell lines. Images were captured using Zeiss LSM 780-NLO confocal microscope. CHD7 (red), actin filaments (green) and nuclei (blue). Bar: 20 µm.

Figure S7


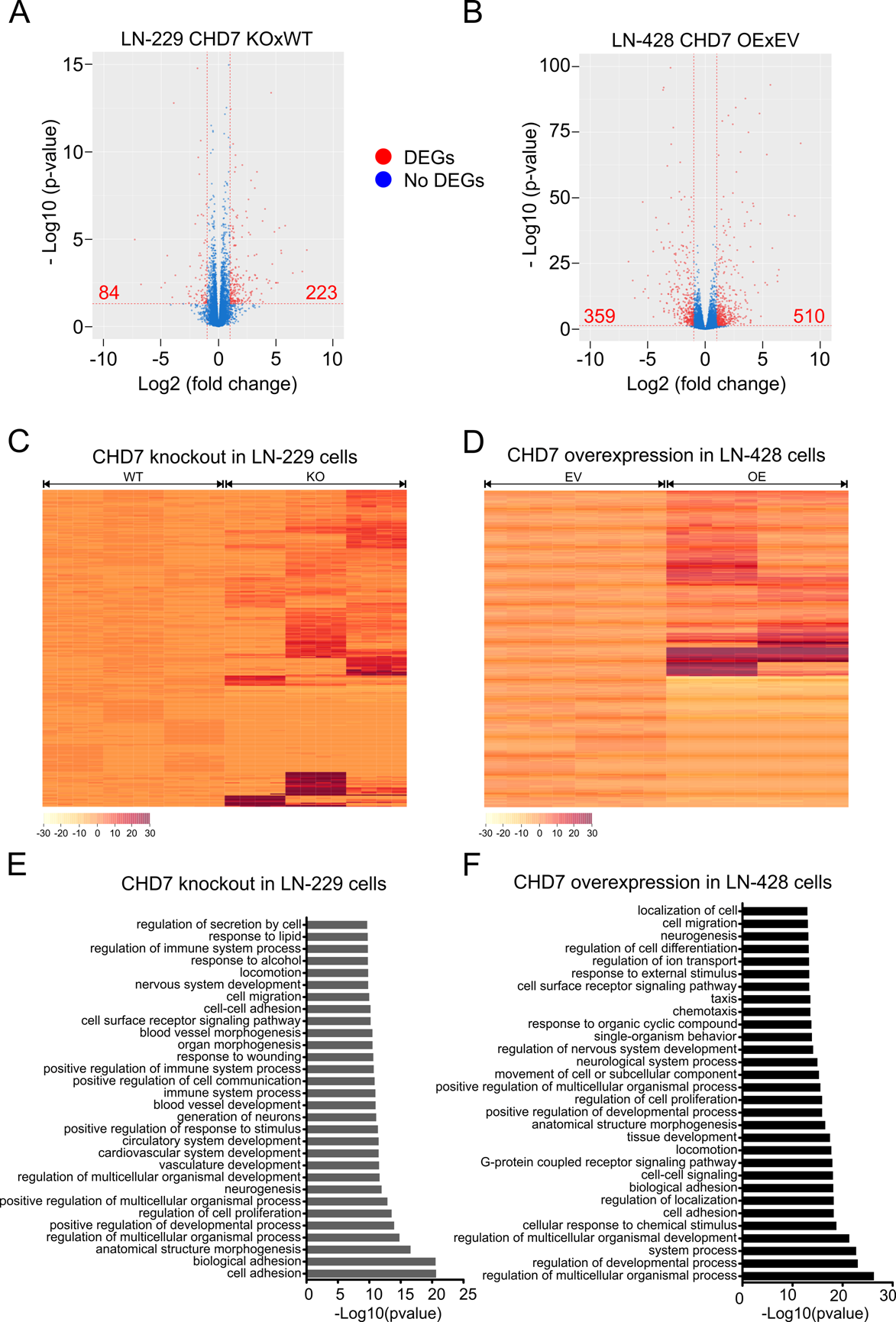


**Figure S7: Modulation in CHD7 levels promotes changes in the transcriptome of glioblastoma cells. (A)** and **(B)** Volcano plots representation of the RNA-seq data, indicating the genes which are significantly and differentially expressed between LN-229 KOxWT and LN-428 OExEV, respectively. Each red dot indicates a gene significantly modulated. The x-axis shows the log2 fold change and the y-axis shows the p value expressed in − log10 scale. CHD7 KO altered the expression of 307 genes (223 down and 84 up) in LN-229 cells and CHD7 OE modified the expression of 869 genes (359 down and 510 up) in LN-428 cells (FDR < 0.05; abs(LFC) > 1, case over the control). **(C)** Unsupervised hierarchical clustering of whole-transcriptome RNA-seq data of three independent KO and WT clones. **(D)** Unsupervised hierarchical clustering of whole-transcriptome RNA-seq data of two independent samples of LN-428 EV and OE cell populations. **(E)** and **(F)** Gene ontology analysis indicating the 30 most enriched pathways for the significantly altered genes in the modified cell lines, defined by STRING database.

Figure S8


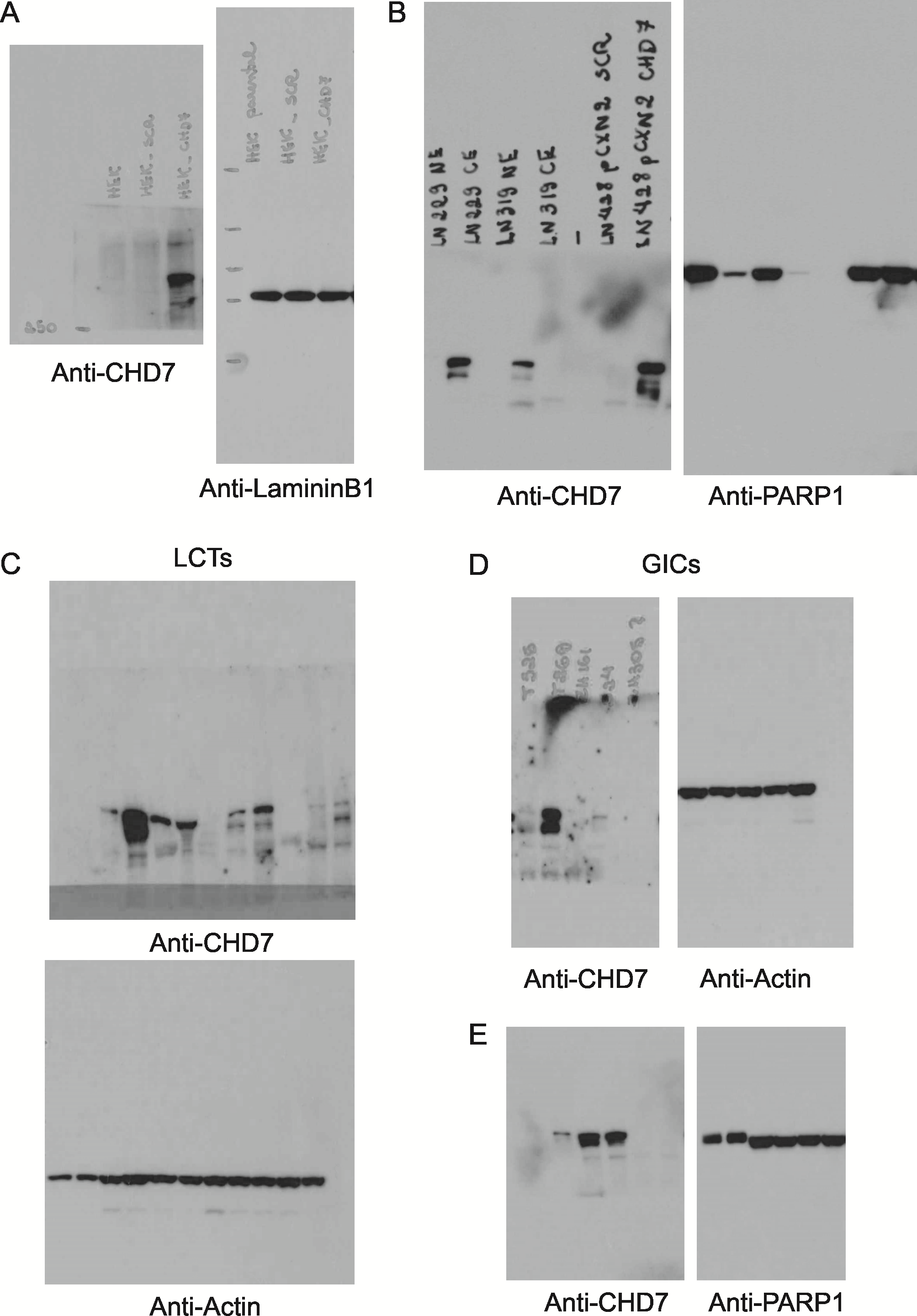


**Figure S8: Original immunoblots used as representative results in the main text. (A)** CHD7 immunoblot confirming the overexpression (OE) in HEK293t cells. LaminB1 was detected in the same membrane as CHD7 to be used as loading control. **(B)** Blot confirming CHD7 nuclear localization using nuclear extracts (NE) and cytoplasmic extracts (CE) of the cell lines LN-229 and LN-319 and we also confirmed LN-428 OE using NE of the empty vector and OE cell populations. In the same membrane, we detected PARP1 signal to confirm the quality of cell fractionation as nuclear marker and loading control for the LN-428 samples. **(C)** Upper image shows CHD7 detection in LTCs using 3-8% pre-cast gel. Due to the great size difference between CHD7 (336KDa) and Actin (42KDa), a parallel 10% gel was loaded in the same conditions to detect the loading control. **(D)** Blots show CHD7 detection in GICs using 3-8% pre-cast gel. As previously performed for the LTCs, a parallel 10% gel was loaded in the same conditions to detect the loading control. **(E)** Blot confirming LN-229 WT and CHD7_KO cell clones. 293T_CHD7 total extract was used as positive control. In the same membrane, we detected PARP1 as loading control.

**References:**

1. Seystahl, K., Tritschler, I., Szabo, E., Tabatabai, G. & Weller, M. Differential regulation of TGF-β 2-induced, ALK-5-mediated VEGF release by SMAD2/3 versus SMAD1/5/8 signaling in glioblastoma. *Neuro. Oncol.* **17,** 254–265 (2015).

2. Lemke, D. *et al.* Primary çjlioblastoma cultures: Can profiling of stem cell markers predict radiotherapy sensitivity? *J. Neurochem.* **131,** 251–264 (2014).

3. Trombetta-Lima, M. *et al.* Isolation and characterization of novel *RECK* tumor suppressor gene splice variants. *Oncotarget* **6,** (2015).

4. Liao, Y., Smyth, G. K. & Shi, W. The Subread aligner: Fast, accurate and scalable read mapping by seed-and-vote. *Nucleic Acids Res.* **41,** (2013).

5. Robinson, M. D., McCarthy, D. J. & Smyth, G. K. edgeR: A Bioconductor package for differential expression analysis of digital gene expression data. *Bioinformatics* **26,** 139–140 (2009).

6. Ran, F. A. *et al.* Genome engineering using the CRISPR-Cas9 system. *Nat. Protoc.* **8,** 2281–308 (2013).
